# Evidence of White Matter Neuroinflammation in Myalgic Encephalomyelitis/Chronic Fatigue Syndrome: A Diffusion‐Based Neuroinflammation Imaging Study

**DOI:** 10.1002/hbm.70505

**Published:** 2026-03-16

**Authors:** Qiang Yu, Kiana Kothe, Richard A. Kwiatek, Peter Del Fante, Anya Bonner, Vince D. Calhoun, Zack Y. Shan

**Affiliations:** ^1^ Thompson Institute University of the Sunshine Coast Birtinya Queensland Australia; ^2^ Tri‐Institutional Center for Translational Research in Neuroimaging and Data Science (TReNDS), Georgia State University, Georgia Institute of Technology Emory University Atlanta Georgia USA

**Keywords:** diffusion tensor imaging, diffusion‐based neuroinflammation imaging model, myalgic encephalomyelitis/chronic fatigue syndrome, neuroinflammation, white matter microstructure

## Abstract

Myalgic encephalomyelitis/chronic fatigue syndrome (ME/CFS) is a debilitating disorder with suspected neuroinflammatory pathophysiology. However, previous diffusion tensor imaging (DTI) studies have reported inconsistent white matter abnormalities in ME/CFS, and specific white matter inflammatory changes remain poorly characterised. This study employed an advanced diffusion‐based neuroinflammation imaging (NII) model to investigate white matter neuroinflammation in ME/CFS. Diffusion MRI data from 67 ME/CFS patients (median age, 38; and 54 women) and 67 rigorously matched healthy controls (HCs) (median age 38; and 52 women) were analysed. Seven NII‐derived metrics were computed: hindered water ratio (NII‐HR), restricted fraction (NII‐RF), fibre fraction (NII‐FF), axial diffusivity (NII‐AD), radial diffusivity (NII‐RD), mean diffusivity (NII‐MD) and fractional anisotropy (NII‐FA). Conventional DTI metrics were also calculated. Tract‐based spatial statistics were used to perform voxel‐wise group comparisons, and multiple regression analysis was conducted to examine the relationship between NII/DTI metrics and clinical measures of mental health, physical health, sleep quality, disability, disease severity and disease duration. Compared to HCs, ME/CFS patients exhibited widespread white matter abnormalities, including significantly lower NII‐HR and NII‐RF, and significantly higher NII‐FF, NII‐AD, NII‐MD and NII‐FA across association, commissural and projection fibres. Additionally, some regions showed decreased NII‐AD and NII‐MD in ME/CFS. Lower NII‐RF, NII‐AD and NII‐MD in ME/CFS were significantly associated with worse mental health, while lower NII‐RF was also associated with a higher level of disability. Among ME/CFS patients, higher NII‐FF was associated with lower disease severity. Conventional DTI showed minimal group differences and no significant clinical associations. This study provides in vivo evidence of white matter neuroinflammation in ME/CFS, characterised by cerebral edema (reduced NII‐HR), cellular infiltration (reduced NII‐RF) and axonal reorganisation (increased NII‐FF). This suggests NII‐derived indices may serve as sensitive biomarkers for neuroinflammation in ME/CFS.

## Graphical abstract

Diffusion‐based neuroinflammation imaging (NII) reveals widespread white matter abnormalities in ME/CFS patients, undetected by conventional DTI. NII metrics associate with mental health, disability and disease severity, providing novel evidence of neuroinflammation and highlighting NII's potential as a biomarker in ME/CFS.

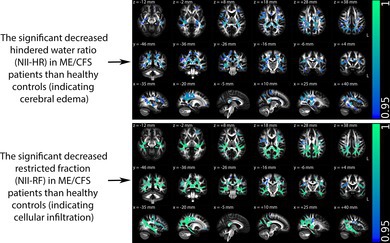

## 1 Introduction

Myalgic encephalomyelitis/chronic fatigue syndrome (ME/CFS) is a debilitating and complex multisystem illness characterised by persistent fatigue, post‐exertional malaise, cognitive impairment, sleep disturbances and autonomic dysfunction (Cortes Rivera et al. 2019; Fukuda et al. 1994). Despite decades of research, the biological mechanisms underlying ME/CFS remain poorly understood, and no definitive diagnostic biomarkers have been established. Neuroinflammation in ME/CFS has been postulated and supported by elevated pro‐inflammatory cytokine levels (Montoya et al. 2017), microglial activation (Nakatomi et al. 2014), and metabolite abnormalities (Mueller et al. 2020). Neuroimaging studies have increasingly implicated the central nervous system in ME/CFS, particularly white matter abnormalities that may reflect neuroinflammatory processes (Barnden et al. 2011; Shan et al. 2018; Nakatomi et al. 2014).

Diffusion tensor imaging (DTI) has been widely used to assess white matter microstructure, revealing alterations in ME/CFS patients compared to healthy controls (HCs), including changes in fractional anisotropy (FA), axial diffusivity (AD), radial diffusivity (RD) and mean diffusivity (MD) (Zeineh et al. 2015; Kimura et al. 2019, 2023; Thapaliya et al. 2021; Josev et al. 2023). However, these findings have been inconsistent across studies, likely due to the inclusion of heterogeneous patient subgroups. We recently (Yu et al. 2025) highlighted the heterogeneities of ME/CFS by identifying distinct white matter alteration patterns between post‐infectious ME/CFS (PI‐ME/CFS) and gradual onset ME/CFS (GO‐ME/CFS) using DTI, with increased AD in PI‐ME/CFS and decreased AD in GO‐ME/CFS compared to HCs, respectively. The increased AD in PI‐ME/CFS was associated with worse physical health, while decreased AD in GO‐ME/CFS was related to worse mental health.

Although DTI offers useful markers of microstructural integrity, it lacks biological specificity and cannot demonstrate the contributions of inflammation, edema, or axonal damage. Advanced diffusion models (Oestreich and O'Sullivan 2022) have been developed to address this limitation. One such validated (Wang et al. 2014; Zhan et al. 2018) approach is the diffusion‐based neuroinflammation imaging (NII) model (Wang et al. 2011, 2015, 2019, 2024; Chiang et al. 2014; Samara et al. 2020), which estimates multiple biologically informed indices to quantify inflammation‐related processes, including the hindered water ratio (NII‐HR, indicating extracellular tissue edema), restricted fraction (NII‐RF, indicating inflammation‐related cellularity) and fibre fraction (NII‐FF, indicating apparent axonal density). In addition, the model provides fibre‐compartment diffusivities, axial (NII‐AD), radial (NII‐RD), mean (NII‐MD) and fractional anisotropy (NII‐FA). Unlike conventional DTI metrics, which are biassed by isotropic signals from oedema or cell infiltration, these fibre‐specific diffusivities isolate the anisotropic component of diffusion, thereby improving sensitivity and interpretability for axonal injury and demyelination. These indices offer greater specificity in detecting changes related to extracellular fluid accumulation, cellular infiltration and axonal density, which are hallmarks of neuroinflammation (Ransohoff and Brown 2012). The NII model has been successfully applied to detect neuroinflammation in multiple sclerosis (Wang et al. 2011, 2015), Alzheimer's disease (Wang et al. 2019, 2024), and obesity (Samara et al. 2020). However, to the best of our knowledge, this advanced technique has not yet been used to investigate neuroinflammation in ME/CFS.

In this study, we applied the NII model to a well‐characterised ME/CFS and HCs cohort (Yu et al. 2025) to investigate white matter neuroinflammation. Whereas the previous study explored general white matter impairments using conventional DTI metrics (Yu et al. 2025), the present work focuses specifically on neuroinflammatory processes by leveraging NII‐derived indices. The primary aim of this study was to determine whether ME/CFS is associated with neuroinflammatory signatures in white matter, as reflected in NII‐derived metrics. This study also examined how NII‐derived indices related to clinical measures. We hypothesised that ME/CFS participants would exhibit altered NII‐derived metrics consistent with neuroinflammation, and that these changes would associate with symptom severity.

## 2 Materials and Methods

Ethical approval for this study was obtained from the University of the Sunshine Coast Ethics Committee (A191288) and the study was registered with the Australian New Zealand Clinical Trials Registry (ACTRN12622001095752). All participants provided written informed consent before participation.

The participants included in this study were drawn from the same cohort (76 ME/CFS participants and 67 HCs) as described in Yu et al. (2025), with recruitment procedures as well as inclusion and exclusion criteria previously described in that work. In brief, adult participants (18–65 years old) were recruited. ME/CFS patients were interviewed by two clinicians to confirm a Canadian Consensus Criteria (CCC)‐consistent (Carruthers et al. 2003) diagnosis of ME/CFS. A consensus diagnosis approach was employed to minimise the risk of an ill‐defined disease cohort. The study intentionally recruited HCs with sedentary lifestyles (< 60 min in moderate or high‐intensity activity [i.e., exercise] per week) to reduce the confounding effects of disease deconditioning (Nijs et al. 2011).

This study rigorously investigated white matter neuroinflammation in ME/CFS patients by (i) employing an appropriate sample size with a 1:1 ratio of patients to HCs, and (ii) providing the associated metadata and symptom scores on Zenodo (http://doi.org/10.5281/zenodo.16916830). All eligible HCs (*n* = 67) were retained to optimise statistical power. To avoid group‐size imbalance and maintain a 1:1 ratio of patients to HCs, nine ME/CFS participants (five PI‐ME/CFS and four GO‐ME/CFS participants, respectively) from the pooled cohort in Yu et al. (2025) were excluded to match the HCs. This ensured that there were no significant group differences in age, sex, body mass index (BMI), metabolic equivalents (MET) rate or MRI scan time, which was essential for reducing potential confounding in the imaging analyses.

### 2.1 Clinical Measures

Participants completed standardised questionnaires (Shan et al. 2022), namely Hospital Anxiety and Depression Scale (HADS), 36‐item Short‐Form (SF‐36) Health Survey, Pittsburgh Sleep Quality Index (PSQI), and Bell's Disability Scale (BDS) questionnaires, assessing mental (MCS) and physical (PCS) health, overall sleep quality and disability level. The disease severity and disease duration of ME/CFS patients were evaluated by two clinicians. The MET rate of the task from the Actigraph data was calculated to measure the activity level for each participant. Missing values in MET for seven HCs and four ME/CFS patients, and depression and anxiety scores for one HC were imputed using age‐specific groups (Howell 2007). The same imputation strategy was applied in the current analysis to maintain methodological consistency.

### 2.2 MRI Acquisition

All imaging was performed on a 3T Skyra MRI scanner (Germany, Erlangen) with a 64‐channel head coil. The diffusion MRI data were acquired using a multiband EPI sequence (72 slices, multiband factor 3, dimension = 114 × 114, voxel size 2 × 2 × 2 mm^3^, TR/TE = 4500/123 ms, bipolar diffusion scheme with two diffusion weightings of *b* = 1000 and *b* = 2500 s/mm^2^ with non‐colinear diffusion directions 27 and 62, respectively, and eight volumes of *b* = 0 s/mm^2^ and phase encoding direction = anterior to posterior). Six volumes of *b* = 0 s/mm^2^ with the opposite phase encoding direction in posterior to anterior are also collected for the eddy current correction.

### 2.3 DTI Processing

The standard FSL FDT (FMRIB's Diffusion Toolbox) (Smith et al. 2004) pipeline was used to correct eddy current, motion, and susceptibility distortions using eddy, TOPUP, and eddy_openmp tools. Eddy quality control (qc) tool was used to perform the quality control both at the single subject and group level. The diffusion tensor model was then fitted using ‘dtifit’ to derive DTI‐FA, DTI‐MD, DTI‐AD and DTI‐RD maps. Each participant's DTI‐FA image was nonlinear registered into Montreal Neurological Institute (MNI) 152 standard space using tract‐based spatial statistics (TBSS) (Smith et al. 2006). The FSL_HCP1065 FA 1 × 1 × 1 mm standard‐space image was used as the target. The same DTI dataset and preprocessing pipeline were used in the present study to ensure consistency across analyses.

### 2.4 NII Processing

The NII models the diffusion MRI signal as a linear combination of multiple anisotropic diffusion tensors and a spectrum of isotropic diffusion components as follows (Wang et al. 2011, 2015, 2019, 2024; Chiang et al. 2014; Samara et al. 2020):

(1) $$S_{k}=\sum_{i=1}^{N_{\text{Aniso}}}f_{i}e^{-\left|\vec{b_{k}}\right|\lambda_{⊥i}}e^{-\left|\vec{b_{k}}\right|\left(\lambda_{∥i}-\lambda_{⊥i}\right)\cos^{2}\psi_{\mathrm{ik}}}+\int_{a}^{b}f\left(D\right)e^{-\left|\vec{b_{k}}\right|D}\mathrm{dD},\left(k=1,2,3,\ldots ,K\right),$$

where $S_{k}$ and $\left|\vec{b_{k}}\right|$ are the signal and *b*‐value of the *k*th diffusion gradient; $N_{\text{Aniso}}$ is the number of anisotropic tensors; $\psi_{\mathrm{ik}}$ the angle between the principal direction of the *i*th anisotropic tensor and the *k*th diffusion gradient; $\lambda_{∥i}$ and $\lambda_{⊥i}$ are the AD and RD of the *i*th anisotropic tensor; $f_{i}$ is the signal intensity fraction for the *i*th anisotropic tensor; and *a* and *b* are the low and high diffusivity limits for the isotropic diffusion spectrum $f\left(D\right)$.

The solving of Equation (1) follows the same methodology as described in Wang et al. (2011), except for the parameter estimation method. In this study, a modified hybrid Nelder–Mead simplex search and particle swarm optimisation (MH‐NMSS‐PSO) algorithm (Yu et al. 2022) was employed instead of the regularised non‐negative least‐squares (RNNLS) optimisation. The MH‐NMSS‐PSO approach ensures convergence to the global minimum without requiring multiple runs with randomly initialised values. In contrast, the RNNLS optimisation relies on repeated executions with different initial values to achieve consistent results by means of generalised pattern search.

By solving the NII model, the following NII‐derived indices were obtained: (1) NII‐HR: NII‐derived hindered water ratio, is the hindered fraction of non‐restricted isotropic diffusion (0.3 < *D* ≤ 2.5 μm^2^/ms); (2) NII‐RF: NII‐derived hindered fraction of restricted isotropic diffusion (*D* ≤ 0.3 μm^2^/ms); (3) NII‐FF: NII‐derived fibre fraction; (4) NII‐AD: NII‐derived axial diffusivity; (5) NII‐RD: NII‐derived radial diffusivity; (6) NII‐MD: NII‐derived mean diffusivity; (7) NII‐FA: NII‐derived fractional anisotropy. The same nonlinear deformations obtained from the DTI‐FA image registration to MNI 152 space were applied to the NII‐derived indices to enable group‐level voxel‐wise analysis in standard space.

### 2.5 Statistical Analysis

Statistical methods including group comparisons and multiple regression models were consistent with Yu et al. (2025) to allow direct comparison between NII‐ and DTI‐derived metrics. The chi‐square test was used to compare categorical variable (sex), and the Wilcoxon rank sum test was used to compare other demographic data and behavioural scores between groups. Group comparisons in each NII/DTI‐derived metrics were performed using general linear models controlling for sex, age, BMI, MET, depression and anxiety scores via FSL's randomise tool (Winkler et al. 2014) with 10,000 permutations at each voxel, and multiple regression analyses were also performed using the randomise tool to examine the associations between NII/DTI‐derived metrics and clinical scores. Note that one ME/CFS participant did not have MCS and PCS scores, and two ME/CFS participants did not have global PSQI scores and were therefore excluded from the corresponding multiple regression analysis. All statistical tests were two‐tailed, and family‐wise error (FWE) with a threshold‐free cluster enhancement (TFCE) corrected *p*‐value < 0.05 was used to indicate significant differences.

## 3 Results

### 3.1 Participant Characteristics

**FIGURE 1:**
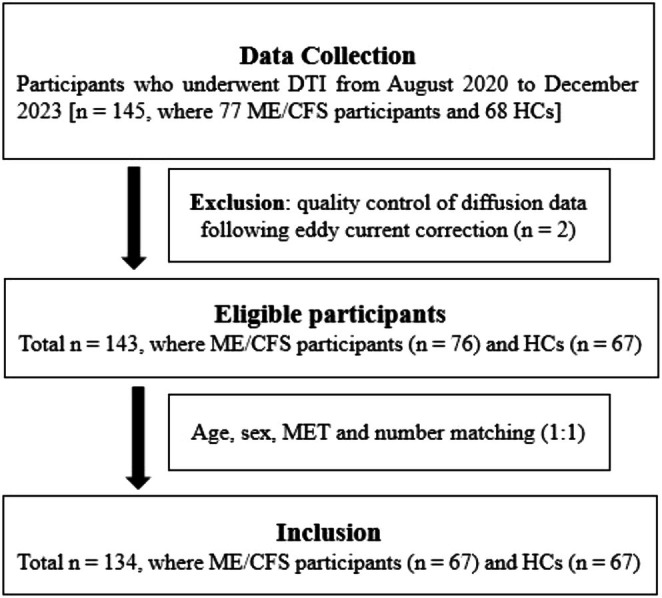
Flowchart of participants included in the study. A total of 134 participants who underwent diffusion tensor imaging (DTI) were included in the study.

DTI data from 134 participants were analysed, including 67 participants with ME/CFS (median age, 38; and 54 women) and 67 HCs (median age, 38; and 52 women) (Figure 1). The demographic data and behavioural scores are reported in Table 1. There are no significant differences in sex (*p* = 0.671), age (*p* = 0.180), BMI (*p* = 0.656), MET rate (*p* = 0.059) or MRI scan time (*p* = 0.931) between the ME/CFS patients and HCs. Compared to ME/CFS, the HCs showed reduced HADS anxiety (*p* < 0.001) and depression (*p* < 0.001). The HCs have increased SF‐36 mental health (*p* < 0.001) and physical health (*p* < 0.001). In addition, the HCs have better overall sleep quality (*p* < 0.001) and lower level of disability (*p* < 0.001) than the patient's group.

**TABLE 1:** Demographic and behavioural information of the study participants.

Characteristics	ME/CFS participants (*n* = 67)	Healthy controls (HCs, *n* = 67)	*p*
Female/male	54 (81%)/13 (19%)	52 (78%)/15 (22%)	0.671
Age (years)	38 (24–65)	38 (19–65)	0.180
BMI (kg/m^2^)	25.00 (17.18–34.89)	24.01 (17.36–35.38)	0.656
MET rate	1.29 (1.13–1.74)	1.34 (1–1.89)	0.059
MRI scan time	11.63 (7.80–15.88)	11.47 (7.43–16.20)	0.931
HADS anxiety	8 (0–18)	4 (0–16)	< 0.001
HADS‐depression	6 (1–17)	1 (0–11)	< 0.001
SF‐36—MCS	44.93 (23.34–58.09)	55.22 (37.18–62.46)	< 0.001
SF‐36—PCS	35.97 (15.83–57.23)	60.24 (35.43–68.30)	< 0.001
Global PSQI	10 (2–17)	4 (0–14)	< 0.001
BDS	40 (10–90)	100 (40–100)	< 0.001
Disease severity	3 (1–5)	n/a	n/a
Disease duration (years)	10 (0.75–45)	n/a	n/a

*Note:* The data are presented as median (minimum–maximum). Continuous variables were compared using the Wilcoxon rank sum test, and reported *p*‐values were based on *t*‐tests. Categorical variable (sex) was compared using the *χ* ^2^‐test. MRI scan times were reported in 24‐h decimal format, such as 16.20 represents 4:12 PM. Disease severity score 1 is referred to as Mild, score 2 is referred to as Mild–Moderate, score 3 is referred to as Moderate, score 4 is referred to as Moderate–Severe, and score 5 is referred to as Severe.

Abbreviations: BDS, Bell's Disability Scale; BMI, body mass index; HADS, Hospital Anxiety and Depression Scale; MCS, mental component summary; ME/CFS, myalgic encephalomyelitis/chronic fatigue syndrome; MET, metabolic equivalents (one MET is defined as the energy used in resting or sitting still); PCS, physical component summary; PSQI, Pittsburgh Sleep Quality Index; SF‐36, 36‐item Short‐Form.

As a complementary investigation, we also applied our methodology to the three‐group analysis of participants, namely post‐infectious ME/CFS (PI‐ME/CFS, *n* = 43), gradual onset ME/CFS (GO‐ME/CFS, *n* = 33) and HCs (*n* = 58), matched for age, sex and MET, as reported in appendix B of Yu et al. (2025). This included NII‐derived metrics, which were compared to enable direct comparisons between NII‐ and DTI‐derived measures. The corresponding results are presented in Appendix A of the Supporting Information. As both PI‐ME/CFS and GO‐ME/CFS groups showed the same pattern of neuroinflammation‐related alterations across NII‐derived metrics, namely lower NII‐RF and higher NII‐FF when compared with HCs, the patient group was not separated by onset for the main findings in this study.

### 3.2 Group Comparison of NII‐Derived Metrics

**FIGURE 2:**
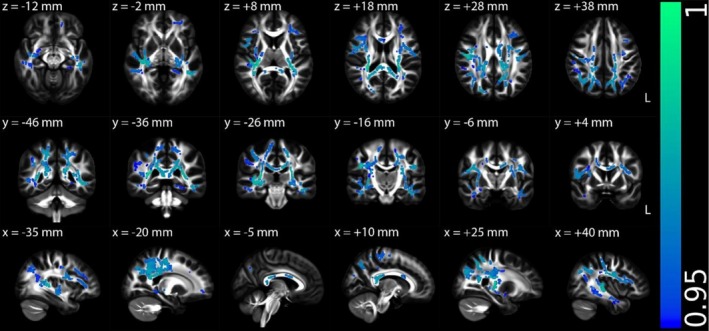
Tract‐based spatial statistics (TBSS) results comparing diffusion‐based neuroinflammation imaging‐derived hindered water ratio (NII‐HR) between the healthy control (*n* = 67) and ME/CFS (*n* = 67) participant groups. Results are displayed in Montreal Neurological Institute (MNI) 152 standard space based on the reference FSL_HCP1065 fractional anisotropy 1 × 1 × 1 mm standard‐space image. The top row shows the results from six different axial slices, where the *z*‐coordinates in MNI space from left to right are *z* = −12, −2, 8, 18, 28 and 38 mm, respectively. The middle row shows the results from six different coronal slices, where the *y*‐coordinates in MNI space from left to right are *y* = −46, −36, −26, −16, −6 and 4 mm, respectively. The bottom row shows the results from six different sagittal slices, where the *x*‐coordinates in MNI space from left to right are *x* = −35, −20, −5, 10, 25 and 40 mm, respectively. Blue–green clusters show the significant decreased NII‐HR in ME/CFS participants. The colour bar represents 1 − *p* values (a dimensionless probability measure), with higher values indicating greater levels of statistical significance.

**FIGURE 3:**
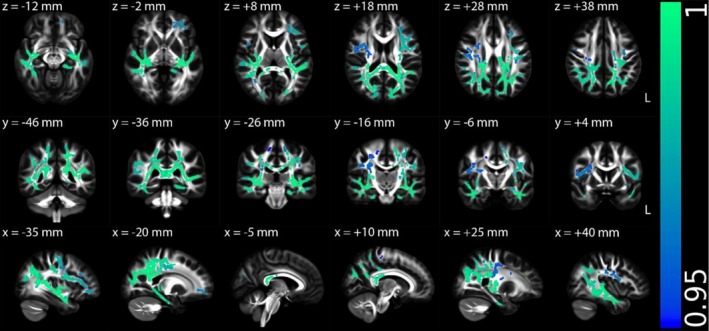
Tract‐based spatial statistics (TBSS) results comparing diffusion‐based neuroinflammation imaging‐derived hindered fraction of restricted isotropic diffusion (NII‐RF) between the healthy control (*n* = 67) and ME/CFS (*n* = 67) participant groups. Results are displayed in Montreal Neurological Institute (MNI) 152 standard space based on the reference FSL_HCP1065 fractional anisotropy 1 × 1 × 1 mm standard‐space image. The top row shows the results from six different axial slices, where the *z*‐coordinates in MNI space from left to right are *z* = −12, −2, 8, 18, 28 and 38 mm, respectively. The middle row shows the results from six different coronal slices, where the *y*‐coordinates in MNI space from left to right are *y* = −46, −36, −26, −16, −6 and 4 mm, respectively. The bottom row shows the results from six different sagittal slices, where the *x*‐coordinates in MNI space from left to right are *x* = −35, −20, −5, 10, 25 and 40 mm, respectively. Blue–green clusters show the significant decreased NII‐RF in ME/CFS participants. The colour bar represents 1 − *p* values (a dimensionless probability measure), with higher values indicating greater levels of statistical significance.

**FIGURE 4:**
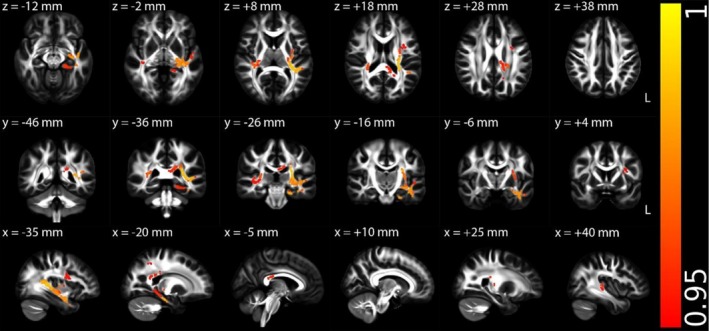
Tract‐based spatial statistics (TBSS) results comparing diffusion‐based neuroinflammation imaging‐derived fibre fraction (NII‐FF) between the healthy control (*n* = 67) and ME/CFS (*n* = 67) participant groups. Results are displayed in Montreal Neurological Institute (MNI) 152 standard space based on the reference FSL_HCP1065 fractional anisotropy 1 × 1 × 1 mm standard‐space image. The top row shows the results from six different axial slices, where the *z*‐coordinates in MNI space from left to right are *z* = −12, −2, 8, 18, 28 and 38 mm, respectively. The middle row shows the results from six different coronal slices, where the *y*‐coordinates in MNI space from left to right are *y* = −46, −36, −26, −16, −6 and 4 mm, respectively. The bottom row shows the results from six different sagittal slices, where the *x*‐coordinates in MNI space from left to right are *x* = −35, −20, −5, 10, 25 and 40 mm, respectively. Red–yellow clusters show the significant increased NII‐FF in ME/CFS participants. The colour bar represents 1 − *p* values (a dimensionless probability measure), with higher values indicating greater levels of statistical significance.

Figures 2, 3, 4, S15–S19 (Appendix B.1 in Supporting Information), and Table 2 illustrate the TBSS results of NII‐derived metrics comparing the HCs and ME/CFS patient groups in MNI 152 standard space. As shown in Figures 2 and 3 and Table 2, the NII‐HR and NII‐RF in the ME/CFS patient group were significantly lower than those in the HCs in several association, commissural and projection fibres, respectively. Figures 4 and S15 (Appendix B.1 in Supporting Information), and Table 2 demonstrate that the NII‐FF and NII‐FA in ME/CFS were significantly higher than those in the HCs in widespread white matter fibre tracts, respectively. Figures S16–S19 (Appendix B.1 in Supporting Information) and Table 2 reveal the regionally heterogeneous pattern of NII‐AD and NII‐MD between ME/CFS and HCs, namely both increased and decreased NII‐AD and NII‐MD were detected in different white matter tracts. There were no significant group differences in TBSS for NII‐RD between ME/CFS and HCs.

**TABLE 2:** Group comparisons of NII‐ and DTI‐derived metrics between the healthy control and ME/CFS participant groups.

Metric	Finding	Significant white matter fibre tracts	Notes
NII‐HR	Lower in ME/CFS	Association fibres (left cingulum hippocampus, SLF and external capsule), commissural fibres (body and splenium of the corpus callosum, and tapetum) and projection fibres (PTR, SCR, left ACR, PCR, PLIC, RLIC, sagittal stratum and stria terminalis) (Figure 2)	Suggests increased extracellular edema
NII‐RF	Lower in ME/CFS	Association fibres (right cingulum cingulate, cingulum hippocampus, SLF, uncinate fasciculus and external capsule), commissural fibres (body and splenium of the corpus callosum, and tapetum) and projection fibres (PTR, SCR, left ACR, PCR, PLIC, RLIC, sagittal stratum and stria terminalis) (Figure 3)	Suggests increased inflammatory cell infiltration
NII‐FF	Higher in ME/CFS	Association fibres (left cingulum hippocampus, left SLF, left uncinate fasciculus and left external capsule), commissural fibres (body and splenium of the corpus callosum and right tapetum) and projection fibres (PTR, left SCR, PCR, PLIC, RLIC, left sagittal stratum and left stria terminalis) (Figure 4)	Suggests increased apparent axonal density
NII‐FA	Higher in ME/CFS	Association fibres (SLF, and right external capsule), commissural fibres (body and splenium of the corpus callosum, and right tapetum) and projection fibres (PTR, right SCR, PCR, PLIC, RLIC, right sagittal stratum and right stria terminalis) (Figure S15)	Largely overlapped with areas of reduced NII‐HR, NII‐RF and increased NII‐FF in ME/CFS
NII‐AD	Higher in ME/CFS	Association fibres (left cingulum hippocampus, left SLF, left uncinate fasciculus and external capsule), commissural fibres (body and splenium of the corpus callosum and right tapetum) and projection fibres (PTR, left SCR, PCR, PLIC, RLIC, sagittal stratum and stria terminalis) (Figure S16)	Largely overlapped with areas of reduced NII‐HR, NII‐RF and increased NII‐FF in ME/CFS
NII‐AD	Lower in ME/CFS	Association fibres (SLF), commissural fibres (body and genu of the corpus callosum) and projection fibres (right cerebellar peduncle, SCR and ACR) (Figure S17)	Opposite direction to increased NII‐AD in other fibres
NII‐MD	Higher in ME/CFS	Association fibres (left cingulum hippocampus, left SLF, left uncinate fasciculus and external capsule), commissural fibres (body and splenium of the corpus callosum, and tapetum) and projection fibres (PTR, SCR, PCR, PLIC, RLIC, sagittal stratum and stria terminalis) (Figure S18)	Largely overlapped with areas of reduced NII‐HR, NII‐RF and increased NII‐FF in ME/CFS
NII‐MD	Lower in ME/CFS	Association fibres (SLF), commissural fibres (body and genu of the corpus callosum) and projection fibres (SCR and ACR) (Figure S19)	Similar tracts as decreased NII‐AD in ME/CFS, and opposite direction to increased NII‐MD in other fibres
DTI‐AD	Higher in ME/CFS	Association fibres (right uncinate fasciculus, and right external capsule), and projection fibres (middle cerebellar peduncle, right sagittal stratum, right stria terminalis and left corticospinal tract) (Figure S20)	
NII‐RD, DTI‐FA, DTI‐MD, and DTI‐RD	No significant group differences		

Abbreviations: ACR, anterior corona radiata; AD, axial diffusivity; DTI, diffusion tensor imaging; FA, fractional anisotropy; FF, fibre fraction; HR, hindered water ratio; MD, mean diffusivity; ME/CFS, myalgic encephalomyelitis/chronic fatigue syndrome; NII, neuroinflammation imaging; PCR, posterior corona radiata; PLIC, posterior limb of internal capsule; PTR, posterior thalamic radiation; RD, radial diffusivity; RF, restricted fraction; RLIC, retrolenticular part of the internal capsule; SCR, superior corona radiata; SLF, superior longitudinal fasciculus.

### 3.3 Group Comparison of DTI‐Derived Metrics

Figure S20 (Appendix B.1 in Supporting Information) and Table 2 illustrate the TBSS DTI‐AD results between the HCs and ME/CFS patient groups in MNI 152 standard space. As shown in Figure S20 and Table 2, the DTI‐AD in the ME/CFS patient group was significantly higher than those in the HCs in several white matter fibre tracts. There were no significant group differences in TBSS for DTI‐FA, DTI‐MD and DTI‐RD between ME/CFS and HCs.

### 3.4 Group Comparison of NII‐ and DTI‐Derived Metrics Without Controlling for Confounding Factors

Without controlling for confounding factors, Figure S23 (Appendix B.3 in Supporting Information) exhibits that the NII‐RF in the ME/CFS patient group was significantly lower than those in the HCs in several association, commissural, and projection fibres. There were no other significant group differences in TBSS for NII‐derived metrics between ME/CFS and HCs when no confounding factors were controlled for. In addition, there were no significant group differences in any DTI‐derived metrics between ME/CFS and HCs that did not control for potential confounding factors.

### 3.5 Multiple Regression Between NII‐Derived Metrics and Clinical Measures

#### 3.5.1 Lower NII‐RF Associated With Worse Mental Health and Increased Disability

**FIGURE 5:**
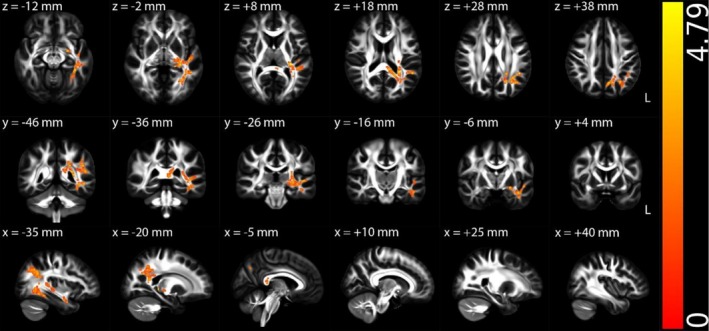
Multiple regression *t*‐statistical results of diffusion‐based neuroinflammation imaging‐derived hindered fraction of restricted isotropic diffusion (NII‐RF) with mental component summary (MCS) among all participants (67 healthy controls and 67 ME/CFS participants). Results are displayed in Montreal Neurological Institute (MNI) 152 standard space based on the reference FSL_HCP1065 fractional anisotropy 1 × 1 × 1 mm standard‐space image. The top row shows the results from six different axial slices, where the *z*‐coordinates in MNI space from left to right are *z* = −12, −2, 8, 18, 28 and 38 mm, respectively. The middle row shows the results from six different coronal slices, where the *y*‐coordinates in MNI space from left to right are *y* = −46, −36, −26, −16, −6 and 4 mm, respectively. The bottom row shows the results from six different sagittal slices, where the *x*‐coordinates in MNI space from left to right are *x* = −35, −20, −5, 10, 25 and 40 mm, respectively. Red–yellow clusters show the significant positive association (FWE corrected *p* < 0.05 with TFCE) of NII‐RF with MCS for all participants. The colour bar represents *t*‐statistical values.

**FIGURE 6:**
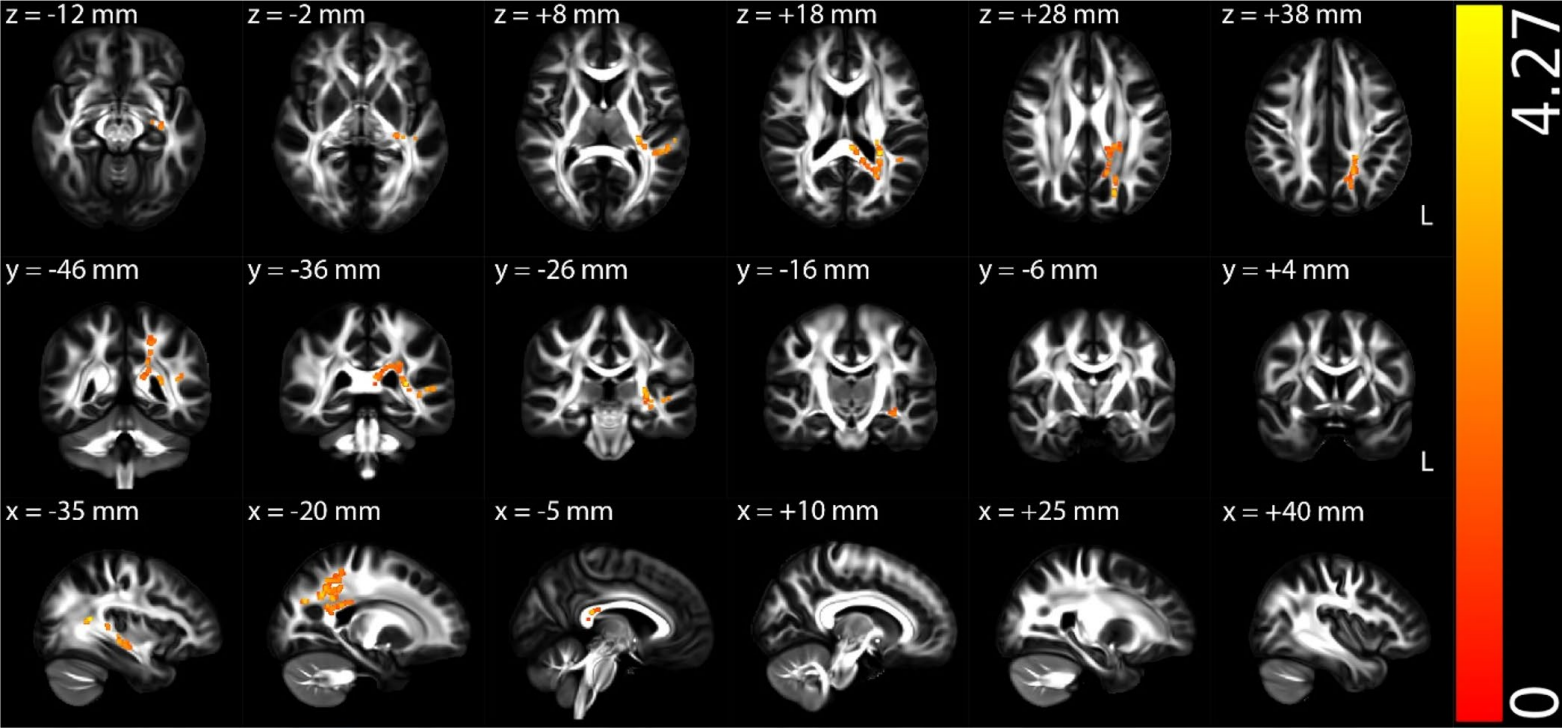
Multiple regression *t*‐statistical results of diffusion‐based neuroinflammation imaging‐derived hindered fraction of restricted isotropic diffusion (NII‐RF) with Bell's disability scale (BDS) among all participants (67 healthy controls and 67 ME/CFS participants). Results are displayed in Montreal Neurological Institute (MNI) 152 standard space based on the reference FSL_HCP1065 fractional anisotropy 1 × 1 × 1 mm standard‐space image. The top row shows the results from six different axial slices, where the *z*‐coordinates in MNI space from left to right are *z* = −12, −2, 8, 18, 28 and 38 mm, respectively. The middle row shows the results from six different coronal slices, where the *y*‐coordinates in MNI space from left to right are *y* = −46, −36, −26, −16, −6 and 4 mm, respectively. The bottom row shows the results from six different sagittal slices, where the *x*‐coordinates in MNI space from left to right are *x* = −35, −20, −5, 10, 25 and 40 mm, respectively. Red–yellow clusters show the significant positive association (FWE corrected *p* < 0.05 with TFCE) of NII‐RF with BDS for all participants. The colour bar represents *t*‐statistical values.

Among all participants (including patients and HCs), significantly positive associations were observed between NII‐RF and MCS or BDS across major white matter tracts (Figures 5 and 6, Table 3). Note that the regions where NII‐RF significantly associated with MCS and BDS largely overlapped with the regions where ME/CFS participants exhibited significantly lower NII‐RF compared to HCs.

**TABLE 3:** Multiple regression between NII or DTI metrics and clinical measures among all participants (including ME/CFS patients and healthy controls).

Metric	*β*	Significant white matter fibre tracts	Notes
NII‐RF	MCS (+)	Association fibres (left SLF, left uncinate fasciculus and left external capsule), commissural fibres (body and splenium of the corpus callosum and left tapetum) and projection fibres (left cerebellar peduncle, left PTR, left PCR, left PLIC, left RLIC, left sagittal stratum and left stria terminalis) (Figure 5)	Largely overlapped with areas of reduced NII‐RF in ME/CFS in group comparison
NII‐RF	BDS (+)	Association fibres (left SLF and left external capsule), commissural fibres (body and splenium of the corpus callosum, and left tapetum) and projection fibres (left PTR, left PCR, left RLIC, left sagittal stratum and left stria terminalis) (Figure 6)	Overlapped with areas of reduced NII‐RF in ME/CFS in group comparison
NII‐AD	MCS (+)	Commissural fibres (body and genu of the corpus callosum) and a projection fibre (ACR) (Figure S21)	Overlapped with areas of reduced NII‐AD in ME/CFS in group comparison
NII‐MD	MCS (+)	Commissural fibres (body and genu of the corpus callosum) and projection fibres (SCR and ACR) (Figure S22)	Overlapped with areas of reduced NII‐MD in ME/CFS in group comparison
Other NII and all DTI metrics	No significant coefficient		

*Note:* *β* are regression coefficients adjusted for confounding factors, (+) and (−) indicate positive and negative coefficients with specific *t*‐scores illustrated in Figures 5, 6, S21 and S22 (Appendix B.2 in Supporting Information).

Abbreviations: ACR, anterior corona radiata; AD, axial diffusivity; BDS, Bell's disability scale; DTI, diffusion tensor imaging; MCS, mental component summary; MD, mean diffusivity; ME/CFS, myalgic encephalomyelitis/chronic fatigue syndrome; NII, neuroinflammation imaging; PCR, posterior corona radiata; PLIC, posterior limb of internal capsule; PTR, posterior thalamic radiation; RF, restricted fraction; RLIC, retrolenticular part of the internal capsule; SCR, superior corona radiata; SLF, superior longitudinal fasciculus.

#### 3.5.2 Higher NII‐FF in ME/CFS Participants Associated With Lower Disease Severity

**FIGURE 7:**
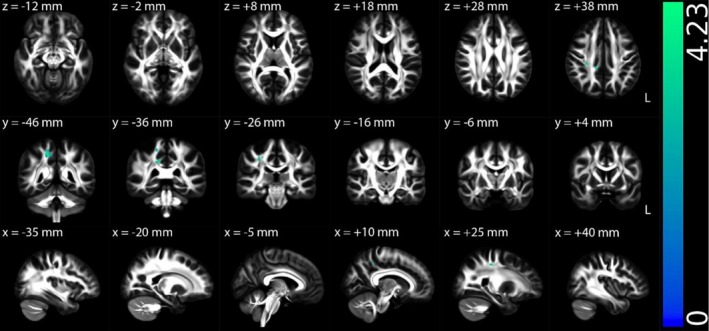
Multiple regression *t*‐statistical results of diffusion‐based neuroinflammation imaging‐derived fibre fraction (NII‐FF) with disease severity for ME/CFS patient group (*n* = 67). Results are displayed in Montreal Neurological Institute (MNI) 152 standard space based on the reference FSL_HCP1065 fractional anisotropy 1 × 1 × 1 mm standard‐space image. The top row shows the results from six different axial slices, where the *z*‐coordinates in MNI space from left to right are *z* = −12, −2, 8, 18, 28 and 38 mm, respectively. The middle row shows the results from six different coronal slices, where the *y*‐coordinates in MNI space from left to right are *y* = −46, −36, −26, −16, −6 and 4 mm, respectively. The bottom row shows the results from six different sagittal slices, where the *x*‐coordinates in MNI space from left to right are *x* = −35, −20, −5, 10, 25 and 40 mm, respectively. Blue–green clusters show the significant negative association (FWE corrected *p* < 0.05 with TFCE) of NII‐FF with disease severity for ME/CFS patient group. The colour bar represents *t*‐statistical values, and *t*‐values are positive in the significant voxels because of the contrast coding used in the analysis.

For the multiple regressions exclusively conducted on the ME/CFS patient group, a significantly negative association was observed between NII‐FF and disease severity in one association fibre (right superior longitudinal fasciculus) and a projection fibre (right posterior corona radiata) (Figure 7 and Table 4). Note that the right superior longitudinal fasciculus did not overlap with areas of higher NII‐FF in ME/CFS in the group comparison, but the right posterior corona radiata showed overlap.

**TABLE 4:** Multiple regression between NII or DTI metrics and clinical measures for ME/CFS patient group only.

Metric	*β*	Significant white matter fibre tracts	Notes
NII‐FF	Disease severity (−)	An association fibre (right SLF) and a projection fibre (right PCR) (Figure 7)	Right SLF did not overlap with areas of higher NII‐FF in ME/CFS in group comparison, but right PCR did overlap
Other NII and all DTI metrics	No significant coefficient		

*Note:* *β* are regression coefficients adjusted for confounding factors, (+) and (−) indicate positive and negative coefficients with specific *t*‐scores illustrated in Figure 7.

Abbreviations: DTI, diffusion tensor imaging; FF, fibre fraction; ME/CFS, myalgic encephalomyelitis/chronic fatigue syndrome; NII, neuroinflammation imaging; PCR, posterior corona radiata; SLF, superior longitudinal fasciculus.

#### 3.5.3 Lower NII‐AD and NII‐MD Associated With Worse Mental Health

Among all participants (including patients and HCs), significantly positive associations were observed between NII‐AD and MCS in commissural fibres (body and genu of the corpus callosum) and a projection fibre (anterior corona radiata) (Figure S21 [Appendix B.2 in Supporting Information] and Table 3). In addition, significantly positive associations were also observed between NII‐MD and MCS in commissural fibres (body and genu of the corpus callosum) and projection fibres (superior corona radiata and anterior corona radiata) (Figure S22 [Appendix B.2 in Supporting Information] and Table 3). Note that the regions where NII‐AD and NII‐MD significantly associated with MCS overlapped with the regions where ME/CFS participants exhibited significantly lower NII‐AD and NII‐MD compared to HCs, respectively.

There were no other significant associations between NII‐derived metrics and clinical measures. In addition, there were no significant associations between DTI‐derived metrics and clinical measures.

### 3.6 Quality of Fitting

Figure S24 (Appendix B.4 in Supporting Information) demonstrates the normalised mean square error map of fitting diffusion MRI signal by solving the NII model using the MH‐NMSS‐PSO algorithm.

## 4 Discussion

This study provides novel evidence of white matter neuroinflammation in ME/CFS using a validated (Wang et al. 2014; Zhan et al. 2018) diffusion‐based NII model. The findings reveal widespread alterations in multiple NII‐derived metrics across white matter fibres, suggesting complex inflammatory processes affecting white matter microstructure in ME/CFS patients.

This study revealed significantly reduced NII‐HR and NII‐RF in ME/CFS patients compared to HCs. NII‐HR reflects hindered extracellular diffusion that may be affected by edema. This edematous state was particularly prominent in the corpus callosum, superior longitudinal fasciculus, and posterior thalamic radiation, regions critical for interhemispheric communication, executive function and sensory integration. The spatial distribution of edema may explain the characteristic cognitive ‘brain fog’ and sensory processing difficulties in ME/CFS. NII‐RF reflects cellularity related to restricted isotropic diffusion, which may correspond to increased inflammatory cell infiltration. The co‐localisation of reduced NII‐HR and NII‐RF in regions such as the left cingulum, hippocampus and uncinate fasciculus, key components of the limbic system, suggests that neuroinflammation particularly affects emotion regulation and memory circuits. These findings are consistent with prior evidence of neuroinflammation in ME/CFS. For example, elevated microglial activation observed via positron emission tomography imaging (Nakatomi et al. 2014), and altered metabolite profiles detected by MR spectroscopy (Mueller et al. 2020; VanElzakker et al. 2019). In particular, elevated choline levels have been linked to neuroinflammation, reflecting both glial activation and increased blood–brain barrier permeability (Albrecht et al. 2016). Reduced NII‐RF, linked to cellularity changes, confirm histopathological reports of immune cell infiltration in ME/CFS (Mandarano et al. 2020). The observed higher NII‐FF in ME/CFS patients further supports the presence of neuroinflammatory processes, where the increased NII‐FF may reflect neuroinflammatory‐driven axonal swelling, gliosis (Nakatomi et al. 2014; Saucier et al. 2023), or reorganisation of axonal fibres (Aung et al. 2013). These increases could also reflect compaction or decreased tortuosity of axonal bundles, a response that has been observed in inflammatory or metabolic stress states where extracellular space shrinks and glial processes encroach on axonal domains (Syková and Nicholson 2008; Garcia‐Hernandez et al. 2022).

Interestingly, both increased and decreased NII‐AD and NII‐MD were detected in different white matter tracts, suggesting heterogeneous underlying pathophysiology. This bidirectional pattern mirrors the previous DTI study (Yu et al. 2025), which revealed opposite directional axial diffusivity (DTI‐AD) changes in post‐infectious and gradual onset ME/CFS subtypes. Even without stratifying by onset, the present analysis reflects this complexity, reinforcing the hypothesis that ME/CFS is a heterogeneous condition characterised by spatially distinct white matter impairments. The regions showing increased NII‐AD and NII‐MD largely overlapped with areas of reduced NII‐HR and NII‐RF, suggesting that inflammation‐related edema and cellular infiltration may increase overall diffusivity in these regions.

Notably, significant associations between NII‐derived metrics and clinical measures, predominantly present in ME/CFS participants, such as the mental health score (MCS), the disability level (BDS) and disease severity, were observed. For example, lower NII‐RF associated with worse mental health and higher levels of disability, and overlapped with ROIs that exhibited group‐level reductions in NII‐RF. Lower RF reflects more inflammatory cellularity, these positive associations imply that less cellular infiltration is associated with better mental health and better function. Anatomically, overlap in callosal, tapetal and posterior thalamic radiation territories connects neuroinflammatory load to networks for interhemispheric integration and thalamic‐cortical relay, circuits linked to fatigue, cognitive efficiency and mood in other disorders (Finke et al. 2015). This supports the hypothesis that neuroinflammatory processes are not only present in ME/CFS but may also relate to symptom severity. Similarly, a negative association between NII‐FF and disease severity suggests that reduced apparent axonal density in the right superior longitudinal fasciculus and right posterior corona radiata may accompany worsening clinical presentation. Lower NII‐FF in more severe patients suggests reduced apparent axonal density or greater dispersion in frontoparietal attention and sensorimotor pathways (Catani and De Schotten 2008). This within group effect complements the group average NII‐FF elevation: on average, patients show higher NII‐FF, but the most severely affected show NII‐FF loss in specific tracts. One tract (right posterior corona radiata) also showed group level NII‐FF increases, implying that NII‐FF may initially rise (e.g., reduced dispersion or compartmental re‐weighting) and later fall with more severe disease (axon or packing loss), a pattern compatible with stage dependent pathology. These associations strengthen the clinical relevance of our imaging findings and highlight the potential of NII‐derived metrics as biomarkers.

In contrast, no significant associations were found between DTI‐derived indices and clinical measures, underscoring the added sensitivity and specificity of the NII model for detecting inflammation‐related changes. Traditional DTI may be less suited for characterising cellular and microstructural changes due to its simplistic tensor model and inability to separate inflammation from other tissue processes (Alexander et al. 2007).

This study has some limitations that should be acknowledged. First, although the NII model offers biologically informed metrics, it is still an indirect measure of neuroinflammation and does not differentiate between specific inflammatory cell types or processes. Validation with other neuroinflammation‐specific techniques would strengthen the interpretations. Second, this study did not collect biological samples alongside neuroimaging data, preventing an investigation of potential peripheral biomarkers associated with the observed findings. Future research incorporating biological sampling could help identify more accessible and cost‐effective biomarkers, improving clinical translation and facilitating subtype‐specific approaches in resource‐limited settings.

## 5 Conclusion

This study provides evidence of white matter neuroinflammation in ME/CFS using advanced diffusion imaging. The widespread alterations in NII metrics, particularly reduced hindered water ratio and restricted fraction, suggest active inflammatory processes involving edema and cellular infiltration. These findings support neuroinflammation as a key pathological feature of ME/CFS and highlight potential targets for anti‐inflammatory therapeutic interventions. The results advance understanding of ME/CFS pathophysiology and demonstrate the value of specialised neuroimaging techniques for detecting subtle but clinically meaningful brain alterations in this complex condition.

## Funding

This work was supported by the National Health and Medical Research Council of Australia (NHMRC) Ideas Grant Scheme (GNT1184219) and The Judith Jane Mason & Harold Stannett Williams Memorial Foundation (The Mason Foundation) under Grant No. Mason2211.

## Ethics Statement

Ethical approval for this study was obtained from the University of the Sunshine Coast Ethics Committee (A191288) and the study was registered with the Australian New Zealand Clinical Trials Registry (ACTRN12622001095752).

## Consent

All participants provided written informed consent before participation.

## Conflicts of Interest

The authors declare no conflicts of interest.

## Supporting information


**Data S1:** Supporting Information.

## Data Availability

The demographics, symptom scores, and preprocessed NII‐ (NII‐HR, NII‐RF, NII‐FF, NII‐AD, NII‐RD, NII‐MD and NII‐FA) and DTI‐ (DTI‐FA, DTI‐MD, DTI‐AD and DTI‐RD) derived metrics of each participant that support the findings of this study are openly available in the Zenodo repository at http://doi.org/10.5281/zenodo.16916830.
