# Correction to “Evidence of White Matter Neuroinflammation in Myalgic Encephalomyelitis/Chronic Fatigue Syndrome: A Diffusion‐Based Neuroinflammation Imaging Study”

**DOI:** 10.1002/hbm.70544

**Published:** 2026-06-02

Yu, Q., K. Kothe, R. A. Kwiatek, et al. 2026. “Evidence of White Matter Neuroinflammation in Myalgic Encephalomyelitis/Chronic Fatigue Syndrome: A Diffusion‐Based Neuroinflammation Imaging Study.” *Human Brain Mapping* 47, no. 4: e70505. https://doi.org/10.1002/hbm.70505.

In the Abstract section, the text “This study provides in vivo evidence of white matter neuroinflammation in ME/CFS, characterised by cerebral edema (reduced NII‐HR), cellular infiltration (reduced NII‐RF), and axonal reorganisation (increased NII‐FF).” was incorrect and should have read: “This study provides in vivo evidence of white matter neuroinflammation in ME/CFS, characterised by cerebral edema (reduced NII‐HR), cell shape change or a chronic depletion of microglia (reduced NII‐RF), and axonal reorganisation (increased NII‐FF).”

In Table 2, the text “Suggests increased inflammatory cell infiltration” was incorrect and should have read: “Suggests cell shape change or a chronic depletion of microglia.” The correct table and its caption are provided below.

**TABLE 2:** Group comparisons of NII‐ and DTI‐derived metrics between the healthy control and ME/CFS participant groups.

Metric	Finding	Significant white matter fibre tracts	Notes
NII‐HR	Lower in ME/CFS	Association fibres (left cingulum hippocampus, SLF and external capsule), commissural fibres (body and splenium of the corpus callosum, and tapetum), and projection fibres (PTR, SCR, left ACR, PCR, PLIC, RLIC, sagittal stratum and stria terminalis) (Figure 2)	Suggests increased extracellular edema
NII‐RF	Lower in ME/CFS	Association fibres (right cingulum cingulate, cingulum hippocampus, SLF, uncinate fasciculus and external capsule), commissural fibres (body and splenium of the corpus callosum, and tapetum), and projection fibres (PTR, SCR, left ACR, PCR, PLIC, RLIC, sagittal stratum and stria terminalis) (Figure 3)	Suggests cell shape change or a chronic depletion of microglia
NII‐FF	Higher in ME/CFS	Association fibres (left cingulum hippocampus, left SLF, left uncinate fasciculus and left external capsule), commissural fibres (body and splenium of the corpus callosum, and right tapetum), and projection fibres (PTR, left SCR, PCR, PLIC, RLIC, left sagittal stratum and left stria terminalis) (Figure 4)	Suggests increased apparent axonal density
NII‐FA	Higher in ME/CFS	Association fibres (SLF, and right external capsule), commissural fibres (body and splenium of the corpus callosum, and right tapetum), and projection fibres (PTR, right SCR, PCR, PLIC, RLIC, right sagittal stratum and right stria terminalis) (Figure S15)	Largely overlapped with areas of reduced NII‐HR, NII‐RF and increased NII‐FF in ME/CFS
NII‐AD	Higher in ME/CFS	Association fibres (left cingulum hippocampus, left SLF, left uncinate fasciculus and external capsule), commissural fibres (body and splenium of the corpus callosum, and right tapetum), and projection fibres (PTR, left SCR, PCR, PLIC, RLIC, sagittal stratum and stria terminalis) (Figure S16)	Largely overlapped with areas of reduced NII‐HR, NII‐RF and increased NII‐FF in ME/CFS
NII‐AD	Lower in ME/CFS	Association fibres (SLF), commissural fibres (body and genu of the corpus callosum), and projection fibres (right cerebellar peduncle, SCR and ACR) (Figure S17)	Opposite direction to increased NII‐AD in other fibres
NII‐MD	Higher in ME/CFS	Association fibres (left cingulum hippocampus, left SLF, left uncinate fasciculus and external capsule), commissural fibres (body and splenium of the corpus callosum, and tapetum), and projection fibres (PTR, SCR, PCR, PLIC, RLIC, sagittal stratum and stria terminalis) (Figure S18)	Largely overlapped with areas of reduced NII‐HR, NII‐RF and increased NII‐FF in ME/CFS
NII‐MD	Lower in ME/CFS	Association fibres (SLF), commissural fibres (body and genu of the corpus callosum), and projection fibres (SCR and ACR) (Figure S19)	Similar tracts as decreased NII‐AD in ME/CFS, and opposite direction to increased NII‐MD in other fibres
DTI‐AD	Higher in ME/CFS	Association fibres (right uncinate fasciculus, and right external capsule), and projection fibres (middle cerebellar peduncle, right sagittal stratum, right stria terminalis and left corticospinal tract) (Figure S20)	
NII‐RD, DTI‐FA, DTI‐MD, and DTI‐RD	No significant group differences		

Abbreviations: ACR, anterior corona radiata; AD, axial diffusivity; DTI, diffusion tensor imaging; FA, fractional anisotropy; FF, fibre fraction; HR, hindered water ratio; MD, mean diffusivity; ME/CFS, myalgic encephalomyelitis/chronic fatigue syndrome; NII, neuroinflammation imaging; PCR, posterior corona radiata; PLIC, posterior limb of internal capsule; PTR, posterior thalamic radiation; RD, radial diffusivity; RF, restricted fraction; RLIC, retrolenticular part of the internal capsule; SCR, superior corona radiata; SLF, superior longitudinal fasciculus.

In the second paragraph of the Discussion section, the text “NII‐RF reflects cellularity related to restricted isotropic diffusion, which may correspond to increased inflammatory cell infiltration.” was incorrect and should have read: “NII‐RF reflects cellularity related to restricted isotropic diffusion. Rather than large, focal inflammatory lesions, ME/CFS may involve a subtle and chronic activation of microglia that causes the cells to change shape (e.g., from ramified to amoeboid) without necessarily increasing their number. The decreased NII‐RF may reflect the shape changes of activated microglia or a chronic depletion of microglia. The reduction in NII‐HR (extracellular water) suggests that the brain is not experiencing vasogenic edema (which would increase extracellular water) but rather a form of cellular swelling or” cytotoxic‐like “edema where the water moves from the extracellular space into the cells or axonal fibres. This matches the observation of higher NII‐AD and NII‐FF, which would occur as the fibres swell and displace the extracellular fluid.”

In the third paragraph of the Discussion section, the text “The regions showing increased NII‐AD and NII‐MD largely overlapped with areas of reduced NII‐HR and NII‐RF, suggesting that inflammation‐related edema and cellular infiltration may increase overall diffusivity in these regions.” was incorrect and should have read: “The regions showing increased NII‐AD and NII‐MD largely overlapped with areas of reduced NII‐HR and NII‐RF, suggesting that inflammation‐related edema and cell shape change or a chronic depletion of microglia may increase overall diffusivity in these regions.”

In the fourth paragraph of the Discussion section, the text “Lower RF reflects more inflammatory cellularity, these positive associations imply that less cellular infiltration is associated with better mental health and better function.” was incorrect and should be removed.

In the Conclusion section, the text “The widespread alterations in NII metrics, particularly reduced hindered water ratio and restricted fraction, suggest active inflammatory processes involving edema and cellular infiltration.” was incorrect and should have read: “The widespread alterations in NII metrics, particularly reduced hindered water ratio and restricted fraction, suggest active inflammatory processes involving edema and cell shape change or a chronic depletion of microglia.”

We apologize for these errors.

