# Supplementary material for: Evidence of White Matter Neuroinflammation in Myalgic Encephalomyelitis/Chronic Fatigue Syndrome: A Diffusion‐Based Neuroinflammation Imaging Study
Source: Hum Brain Mapp. 2026 Mar 16;47(4):e70505. doi: 10.1002/hbm.70505 (PMC13093695; doi:10.1002/hbm.70505)
Supplement: Supplementary file 1 — Data S1: Supporting Information. [file HBM-47-e70505-s001.docx]

**Evidence of white matter neuroinflammation in myalgic encephalomyelitis/chronic fatigue syndrome: a diffusion-based neuroinflammation imaging study**

Qiang Yu^1,*^, Kiana Kothe^1^, Richard A. Kwiatek^1^, Peter Del Fante^1^, Anya Bonner^1^, Vince D. Calhoun^2^, Zack Y. Shan^1^

^1^ Thompson Institute, University of the Sunshine Coast, Birtinya, QLD 4575, Australia

^2^ Tri-institutional Center for Translational Research in Neuroimaging and Data Science (TReNDS), Georgia State University, Georgia Institute of Technology, Emory University, 55 Park Pl NE, 18th Floor, Atlanta, GA 30303, USA

**^*^Corresponding Author:** Qiang Yu (qyu@usc.edu.au)

Kiana Kothe: Kiana.Kothe@research.usc.edu.au

Richard A. Kwiatek: rkwiatek@bigpond.com

Peter Del Fante: peter.delfante@gmail.com

Anya Bonner: abonner@usc.edu.au

Vince D. Calhoun: vcalhoun@gsu.edu

Zack Y. Shan: zshan@usc.edu.au

**Supplementary Material**

**Appendix A**

The Appendix A shows the group comparison results of diffusion-based neuroinflammation imaging (NII)-derived metrics between myalgic encephalomyelitis/chronic fatigue syndrome (ME/CFS) participants with post-infectious (PI-ME/CFS) (n = 43) and gradual onsets (GO-ME/CFS) (n = 33) and healthy controls (HCs) (n = 58) after age, sex and metabolic equivalents (MET) matching. The PI-ME/CFS group consisted of 43 PI-ME/CFS participants (median age, 41; and 37 women) and 58 HCs (median age, 38.5; and 50 women). The GO-ME/CFS group consisted of 33 GO-ME/CFS participants (median age, 40; and 26 women) and 58 HCs (median age, 38.5; and 50 women). All these participants are the same as reported in Appendix B of Yu et al. (2025).

1. **Participant characteristics**

Supplementary Table S1 presents the demographic characteristics and behavioural assessments for PI-ME/CFS (n = 43) and HCs (n = 58). The Chi-Square test was used to assess whether there was a significant difference in sex distribution between PI-ME/CFS and HCs groups, where p < 0.05 was used to indicate significant differences. There is no significant difference in sex (p = 0.982), age (p = 0.069), body mass index (BMI) (p = 0.481), MET rate (p = 0.069), and MRI scan time (p = 0.959) between the PI-ME/CFS patients and HCs. Compared to PI-ME/CFS, the HCs showed reduced Hospital Anxiety and Depression Scale (HADS) anxiety (p < 0.001) and depression (p < 0.001) and increased 36-item Short-Form (SF-36) mental health (p < 0.001) and physical health (p < 0.001). The HCs also have better overall sleep quality (p < 0.001) and lower level of disability (p < 0.001) than the patient’s group.

Supplementary Table S2 demonstrates the demographic characteristics and behavioural assessments for GO-ME/CFS (n = 33) and HCs (n = 58). There is no significant difference in sex (p = 0.359), age (p = 0.122), BMI (p = 0.866), MET rate (p = 0.251), and MRI scan time (p = 0.931) between the GO-ME/CFS patients and HCs. Compared to GO-ME/CFS, the HCs showed reduced HADS anxiety (p < 0.001) and depression (p < 0.001) and increased SF-36 mental health (p < 0.001) and physical health (p < 0.001). The HCs also have better overall sleep quality (p < 0.001) and lower level of disability (p < 0.001) than the patient’s group.

1. **Group comparison between PI-ME/CFS and HCs**

Supplementary Figs. S1-S5 illustrate the tract-based spatial statistics (TBSS) results of NII-derived metrics comparing the HCs and PI-ME/CFS patient groups in MNI 152 standard space. There were no significant group differences in TBSS for NII-HR and NII-RD between PI-ME/CFS and HCs.

**Lower NII-RF and higher NII-FF in PI-ME/CFS participants than HCs**

As shown in Supplementary Fig. S1, the NII-RF in PI-ME/CFS patient group was significantly lower than those in the HCs in the following fibre tracts: association fibres (cingulum hippocampus, superior longitudinal fasciculus, uncinate fasciculus, and external capsule), commissural fibres (body and splenium of corpus callosum and tapetum), and projection fibres (posterior thalamic radiation, superior corona radiata, right anterior corona radiata, posterior corona radiata, posterior limb of internal capsule, retrolenticular part of internal capsule, sagittal stratum, and stria terminalis). Supplementary Fig. S2 demonstrates that the NII-FF in PI-ME/CFS was significantly higher than those in the HCs in the following fibre tracts: association fibres (left cingulum hippocampus, left superior longitudinal fasciculus, left uncinate fasciculus, and left external capsule), commissural fibres (right tapetum), and projection fibres (posterior thalamic radiation, left superior corona radiata, posterior corona radiata, posterior limb of internal capsule, retrolenticular part of internal capsule, sagittal stratum, and stria terminalis).

**Higher NII-AD, NII-FA and NII-MD in PI-ME/CFS participants than HCs**

Supplementary Fig. S3 shows that the NII-AD in PI-ME/CFS was significantly higher than those in the HCs in the following fibre tracts: association fibres (left cingulum hippocampus, superior longitudinal fasciculus, left uncinate fasciculus, and external capsule), commissural fibres (body and splenium of corpus callosum and right tapetum), and projection fibres (posterior thalamic radiation, superior corona radiata, posterior corona radiata, posterior limb of internal capsule, retrolenticular part of internal capsule, sagittal stratum, and stria terminalis). As displayed in Supplementary Fig. S4, the NII-FA in PI-ME/CFS patient group was significantly higher than those in the HCs in the following fibre tracts: association fibres (superior longitudinal fasciculus, and right external capsule), commissural fibres (right tapetum), and projection fibres (posterior thalamic radiation, right superior corona radiata, posterior corona radiata, posterior limb of internal capsule, retrolenticular part of internal capsule, right sagittal stratum, and right stria terminalis). Supplementary Fig. S5 reveals that the NII-MD in PI-ME/CFS was significantly higher than those in the HCs in the following fibre tracts: association fibres (left cingulum hippocampus, superior longitudinal fasciculus, left uncinate fasciculus, and external capsule), commissural fibres (right tapetum), and projection fibres (posterior thalamic radiation, superior corona radiata, posterior corona radiata, posterior limb of internal capsule, retrolenticular part of internal capsule, sagittal stratum, and stria terminalis).

1. **Group comparison between GO-ME/CFS and HCs**

Supplementary Figs. S6-S14 illustrate the TBSS results of NII-derived metrics comparing the HCs and GO-ME/CFS patient groups in MNI 152 standard space.

**Lower NII-HR and NII-RF in GO-ME/CFS participants than HCs**

As shown in Supplementary Fig. S6, the NII-HR in GO-ME/CFS patient group was significantly lower than those in the HCs in the following fibre tracts: association fibres (right cingulum cingulate, right superior longitudinal fasciculus, and right external capsule), commissural fibres (body and splenium of corpus callosum and right tapetum), and projection fibres (posterior thalamic radiation, right superior corona radiata, posterior corona radiata, posterior limb of internal capsule, retrolenticular part of internal capsule, right sagittal stratum, and right stria terminalis). Supplementary Fig. S7 exhibits that the NII-RF in GO-ME/CFS patient group was significantly lower than those in the HCs in the following fibre tracts: association fibres (right cingulum cingulate, left cingulum hippocampus, superior longitudinal fasciculus, uncinate fasciculus, and external capsule), commissural fibres (body and splenium of corpus callosum and left tapetum), and projection fibres (left cerebellar peduncle, posterior thalamic radiation, superior corona radiata, posterior corona radiata, posterior limb of internal capsule, retrolenticular part of internal capsule, sagittal stratum, and stria terminalis).

**Higher NII-FF in GO-ME/CFS participants than HCs**

Supplementary Fig. S8 demonstrates that the NII-FF in GO-ME/CFS was significantly higher than those in the HCs in the following fibre tracts: association fibres (left superior longitudinal fasciculus, and left external capsule), commissural fibres (body and splenium of corpus callosum), and projection fibres (left posterior thalamic radiation, left superior corona radiata, left posterior corona radiata, left posterior limb of internal capsule, left retrolenticular part of internal capsule, left sagittal stratum, and left stria terminalis).

**Lower NII-MD and NII-RD in GO-ME/CFS participants than HCs**

Supplementary Fig. S9 reveals that the NII-MD in GO-ME/CFS was significantly lower than those in the HCs in the following fibre tracts: association fibres (right cingulum cingulate, superior longitudinal fasciculus, superior fronto-occipital fasciculus, and external capsule), commissural fibres (body and genu of corpus callosum), and projection fibres (superior corona radiata, anterior corona radiata, right posterior corona radiata, posterior limb of internal capsule, and anterior limb of internal capsule). As displayed in Supplementary Fig. S10, the NII-RD in GO-ME/CFS was significantly lower than those in the HCs in the following fibre tracts: commissural fibres (corpus callosum), and projection fibres (superior corona radiata, anterior corona radiata, and right retrolenticular part of internal capsule).

**Mixed patterns of NII-AD and NII-FA alterations in GO-ME/CFS: region-specific increases and decreases**

Supplementary Figs. S11 and S12 demonstrate the regionally heterogeneous patterns of NII-AD between GO-ME/CFS and HCs. As shown in Supplementary Fig. S11, the NII-AD in GO-ME/CFS was significantly lower than those in the HCs in the following fibre tracts: association fibres (superior longitudinal fasciculus, superior fronto-occipital fasciculus, and external capsule), commissural fibres (body and genu of corpus callosum), and projection fibres (superior corona radiata, anterior corona radiata, right posterior limb of internal capsule, and anterior limb of internal capsule). Supplementary Fig. S12 represents that the NII-AD in GO-ME/CFS was significantly higher than those in the HCs in the following fibre tracts: association fibres (left superior longitudinal fasciculus), commissural fibres (body and splenium of corpus callosum), and projection fibres (left posterior thalamic radiation, left superior corona radiata, left posterior corona radiata, left posterior limb of internal capsule, retrolenticular part of internal capsule, and stria terminalis).

Supplementary Figs. S13 and S14 exhibit the regionally heterogeneous patterns of NII-FA between GO-ME/CFS and HCs. As shown in Supplementary Fig. S13, the NII-FA in GO-ME/CFS was significantly lower than those in the HCs in projection fibres (left superior corona radiata). Supplementary Fig. S14 illustrates that the NII-FA in GO-ME/CFS was significantly higher than those in the HCs in the following fibre tracts: association fibres (left superior longitudinal fasciculus, and left external capsule), commissural fibres (body and splenium corpus callosum), and projection fibres (posterior thalamic radiation, left superior corona radiata, posterior corona radiata, posterior limb of internal capsule, retrolenticular part of internal capsule, right sagittal stratum, and stria terminalis).

1. **Comparison of NII-AD and DTI-AD alterations**

Comparison of NII-AD and DTI-AD alterations from Appendix B in Yu et al. (2025) revealed markedly different degrees of overlap between PI-ME/CFS and GO-ME/CFS. In PI-ME/CFS, there was substantial spatial concordance between NII-AD and DTI-AD increases, particularly across association, commissural, and projection fibres, including the cingulum hippocampus, superior longitudinal fasciculus, uncinate fasciculus, corpus callosum, posterior thalamic radiation, corona radiata, internal capsule, sagittal stratum, and stria terminalis. In contrast, GO-ME/CFS demonstrated limited overlap between NII-AD and DTI-AD alterations, with DTI-AD changes largely restricted to the corpus callosum, whereas NII-AD exhibited regionally heterogeneous increases and decreases across widespread fibre tracts.

**Appendix B**

The Appendix B shows (i) the group comparison results of NII-AD, NII-RD, NII-MD, NII-FA and DTI-derived metrics (DTI-FA, DTI-MD, DTI-AD, and DTI-RD) between 67 participants with ME/CFS (median age, 38; and 54 women) and 67 HCs (median age, 38; and 52 women) (Fig. 1 in the main text) with controlling sex, age, BMI, MET, depression, and anxiety scores, and (ii) the multiple regression results of NII-AD, NII-RD, NII-MD, NII-FA and DTI-derived metrics (DTI-FA, DTI-MD, DTI-AD, and DTI-RD) with clinical scores, namely mental component summary (MCS), physical component summary (PCS), global Pittsburgh Sleep Quality Index (PSQI), and Bell’s Disability Scale (BDS) scores, for all participants (including 67 HCs and 67 ME/CFS patients) and with disease severity and disease duration for patient group (n = 67) only. All multiple regressions included sex, age, BMI, MET, depression, and anxiety scores as nuisance covariates. (iii) The group comparison results of NII- and DTI-derived metrics between 67 participants with ME/CFS and 67 HCs without controlling sex, age, BMI, MET, depression, and anxiety scores, and (iv) the normalised mean square error map of fitting diffusion MRI signal by solving the NII model using a modified hybrid Nelder-Mead simplex search and particle swarm optimisation (MH-NMSS-PSO) algorithm.

1. **Group comparison between ME/CFS and HCs with controlling for confounding factors**

Supplementary Figs. S15-S20 illustrate the TBSS results of NII-AD, NII-RD, NII-MD, NII-FA and DTI-derived metrics (DTI-FA, DTI-MD, DTI-AD, and DTI-RD) comparing the HCs and ME/CFS patient groups in MNI 152 standard space. There were no significant group differences in TBSS for NII-RD, DTI-FA, DTI-MD, and DTI-RD between ME/CFS and HCs.

**Higher NII-FA in ME/CFS participants than HCs**

As shown in Supplementary Fig. S15, the NII-FA in ME/CFS patient group was significantly higher than those in the HCs in the following fibre tracts: association fibres (superior longitudinal fasciculus, and right external capsule), commissural fibres (body and splenium of corpus callosum, and right tapetum), and projection fibres (posterior thalamic radiation, right superior corona radiata, posterior corona radiata, posterior limb of internal capsule, retrolenticular part of internal capsule, right sagittal stratum, and right stria terminalis).

**Mixed patterns of NII-AD and NII-MD alterations in ME/CFS: region-specific increases and decreases**

Supplementary Figs. S16 and S17 demonstrate the regionally heterogeneous patterns of NII-AD between ME/CFS and HCs. As shown in Supplementary Fig. S16, the NII-AD in ME/CFS was significantly higher than those in the HCs in the following fibre tracts: association fibres (left cingulum hippocampus, left superior longitudinal fasciculus, left uncinate fasciculus, and external capsule), commissural fibres (body and splenium of corpus callosum, and right tapetum), and projection fibres (posterior thalamic radiation, left superior corona radiata, posterior corona radiata, posterior limb of internal capsule, retrolenticular part of internal capsule, sagittal stratum, and stria terminalis). Supplementary Fig. S17 represents that the NII-AD in ME/CFS was significantly lower than those in the HCs in the following fibre tracts: association fibres (superior longitudinal fasciculus), commissural fibres (body and genu of corpus callosum), and projection fibres (right cerebellar peduncle, superior corona radiata, and anterior corona radiata).

Supplementary Figs. S18 and S19 exhibit the regionally heterogeneous patterns of NII-MD between ME/CFS and HCs. As shown in Supplementary Fig. S18, the NII-MD in ME/CFS was significantly higher than those in the HCs in the following fibre tracts: association fibres (left cingulum hippocampus, left superior longitudinal fasciculus, left uncinate fasciculus, and external capsule), commissural fibres (body and splenium of corpus callosum, and tapetum), and projection fibres (posterior thalamic radiation, superior corona radiata, posterior corona radiata, posterior limb of internal capsule, retrolenticular part of internal capsule, sagittal stratum, and stria terminalis). Supplementary Fig. S19 illustrates that the NII-MD in ME/CFS was significantly lower than those in the HCs in the following fibre tracts: association fibres (superior longitudinal fasciculus), commissural fibres (body and genu of corpus callosum), and projection fibres (superior corona radiata, and anterior corona radiata).

**Higher DTI-AD in ME/CFS participants than HCs**

As shown in Supplementary Fig. S20, the DTI-AD in ME/CFS patient group was significantly higher than those in the HCs in the following fibre tracts: association fibres (right uncinate fasciculus, and right external capsule), and projection fibres (middle cerebellar peduncle, right sagittal stratum, right stria terminalis, and left corticospinal tract).

1. **Multiple regression with clinical measures for ME/CFS and HCs**

**Lower NII-AD and NII-MD associated with worse mental health**

Among all participants (including patients and HCs), significantly positive associations were observed between NII-AD and MCS in commissural fibres (body and genu of the corpus callosum) and a projection fibre (anterior corona radiata) (Supplementary Fig. S21). In addition, significantly positive associations were also observed between NII-MD and MCS in commissural fibres (body and genu of the corpus callosum) and projection fibres (superior corona radiata and anterior corona radiata) (Supplementary Fig. S22).

There were no other significant associations between NII-derived metrics and clinical measures. In addition, there were no significant associations between DTI-derived metrics and clinical measures.

1. **Group comparison between ME/CFS and HCs without controlling for confounding factors**

Without controlling for confounding factors, Supplementary Fig. S23 exhibits that the NII-RF in ME/CFS patient group was significantly lower than those in the HCs in the following fibre tracts: association fibres (superior longitudinal fasciculus, right uncinate fasciculus, and external capsule), commissural fibres (body and splenium of corpus callosum, and tapetum), and projection fibres (posterior thalamic radiation, right superior corona radiata, posterior corona radiata, right posterior limb of internal capsule, retrolenticular part of internal capsule, sagittal stratum, and stria terminalis).

There were no other significant group differences in TBSS for NII-derived metrics between ME/CFS and HCs when no confounding factors were controlled for. In addition, there were no significant group differences in any DTI-derived metrics between ME/CFS and HCs that did not control for potential confounding factors.

1. **Quality of fitting**

Supplementary Fig. S24 demonstrates the normalised mean square error map of fitting diffusion MRI signal by solving the NII model using MH-NMSS-PSO algorithm.

Supplementary Table S1: Demographic and behavioural information for post-infectious ME/CFS (PI-ME/CFS) (n = 43) and healthy control (n = 58) groups after age, sex and MET matching.

| Characteristics | PI-ME/CFS  participants (n=43) | Healthy controls  (HCs, n=58) | p values |
| --- | --- | --- | --- |
| Female/male | 37 (86%) / 6 (14%) | 50 (86%) / 8 (14%) | 0.982 |
| Age (years) | 41 (24-65) | 38.50 (19-62) | 0.069 |
| BMI (kg/m^2^) | 25.31 (17.18-34.66) | 23.62 (17.36-35.38) | 0.481 |
| MET rate | 1.27 (1.04-1.74) | 1.31 (1-1.74) | 0.069 |
| MRI scan time | 11.70 (8.05-15.60) | 11.46 (7.43-16.20) | 0.959 |
| HADS-anxiety | 7 (0-15) | 4 (0-16) | < 0.001 |
| HADS-depression | 6 (0-16) | 1 (0-11) | < 0.001 |
| SF-36 - MCS | 45.35 (30.29-58.09) | 55.17 (37.55-62.46) | < 0.001 |
| SF-36 - PCS | 35.83 (15.83-57.23) | 60.30 (35.43-68.30) | < 0.001 |
| Global PSQI | 10 (2-17) | 4 (0-14) | < 0.001 |
| BDS | 40 (20-90) | 100 (40-100) | < 0.001 |
| Disease severity | 3 (1-5) | n/a | n/a |
| Disease duration (years) | 13 (0.75-45) | n/a | n/a |

Note – This table presents the same data as Supplementary Table S5 in Appendix B of Yu et al. (2025), reformatted to report median (minimum–maximum) values and p-values to three decimal places. Continuous variables were compared using the Wilcoxon rank sum test, and reported p-values were based on t-tests. Categorical variable (sex) was compared using the χ^2^-test. MRI scan times were reported in 24-hour decimal format, such as 15.60 represents 3:36 PM. ME/CFS = myalgic encephalomyelitis/chronic fatigue syndrome; BMI = body mass index; MET = metabolic equivalents (one MET is defined as the energy used in resting or sitting still); HADS = Hospital Anxiety and Depression Scale; SF-36 = 36-item Short-Form; MCS = mental component summary; PCS = physical component summary; PSQI = Pittsburgh Sleep Quality Index; BDS = Bell’s Disability Scale. Disease severity score 1 is referred to as Mild, score 2 is referred to as Mild-Moderate, score 3 is referred to as Moderate, score 4 is referred to as Moderate-Severe, and score 5 is referred to as Severe.

Supplementary Table S2: Demographic and behavioural information for gradual onset ME/CFS (GO-ME/CFS) (n = 33) and healthy control (n = 58) groups after age, sex and MET matching.

| Characteristics | GO-ME/CFS  participants (n=33) | Healthy controls  (HCs, n=58) | p values |
| --- | --- | --- | --- |
| Female/male | 26 (79%) / 7 (21%) | 50 (86%) / 8 (14%) | 0.359 |
| Age (years) | 40 (24-62) | 38.50 (19-62) | 0.122 |
| BMI (kg/m^2^) | 24.39 (18.69-34.89) | 23.62 (17.36-35.38) | 0.866 |
| MET rate | 1.30 (1.04-1.72) | 1.31 (1-1.74) | 0.251 |
| MRI scan time | 11.63 (7.80-15.88) | 11.46 (7.43-16.20) | 0.931 |
| HADS-anxiety | 8 (1-18) | 4 (0-16) | < 0.001 |
| HADS-depression | 6 (1-17) | 1 (0-11) | < 0.001 |
| SF-36 - MCS | 44.66 (23.34-56.77) | 55.17 (37.55-62.46) | < 0.001 |
| SF-36 - PCS | 35.97 (27.10-50.68) | 60.30 (35.43-68.30) | < 0.001 |
| Global PSQI | 8.5 (3-15) | 4 (0-14) | < 0.001 |
| BDS | 40 (10-70) | 100 (40-100) | < 0.001 |
| Disease severity | 3 (1-4) | n/a | n/a |
| Disease duration (years) | 8 (1.5-28) | n/a | n/a |

Note – This table presents the same data as Supplementary Table S6 in Appendix B of Yu et al. (2025), reformatted to report median (minimum–maximum) values and p-values to three decimal places. Continuous variables were compared using the Wilcoxon rank sum test, and reported p-values were based on t-tests. Categorical variable (sex) was compared using the χ^2^-test. MRI scan times were reported in 24-hour decimal format, such as 15.60 represents 3:36 PM. ME/CFS = myalgic encephalomyelitis/chronic fatigue syndrome; BMI = body mass index; MET = metabolic equivalents (one MET is defined as the energy used in resting or sitting still); HADS = Hospital Anxiety and Depression Scale; SF-36 = 36-item Short-Form; MCS = mental component summary; PCS = physical component summary; PSQI = Pittsburgh Sleep Quality Index; BDS = Bell’s Disability Scale. Disease severity score 1 is referred to as Mild, score 2 is referred to as Mild-Moderate, score 3 is referred to as Moderate, score 4 is referred to as Moderate-Severe, and score 5 is referred to as Severe.

**Figures**


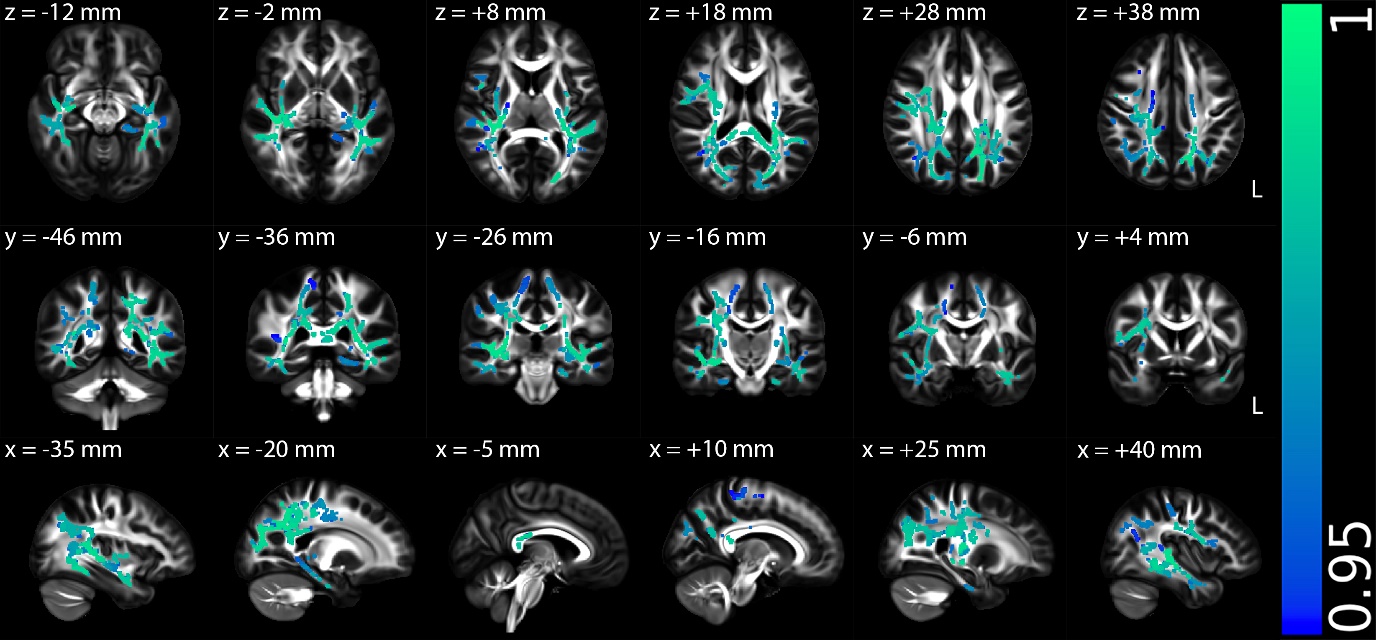


Supplementary Fig. S1: The tract-based spatial statistics (TBSS) results of diffusion-based neuroinflammation imaging-derived hindered fraction of restricted isotropic diffusion (NII-RF) between the healthy control (n = 58) and post-infectious ME/CFS (PI-ME/CFS) (n = 43) participant groups. Results are displayed in Montreal Neurological Institute (MNI) 152 standard space based on the reference FSL_HCP1065 fractional anisotropy 1x1x1mm standard-space image. The top row shows the results from six different axial slices, where the *z*-coordinates in MNI space from left to right are *z* = -12 mm, -2 mm, 8 mm, 18 mm, 28 mm, and 38 mm, respectively. The middle row shows the results from six different coronal slices, where the *y*-coordinates in MNI space from left to right are *y* = -46 mm, -36 mm, -26 mm, -16 mm, -6 mm, and 4 mm, respectively. The bottom row shows the results from six different sagittal slices, where the *x*-coordinates in MNI space from left to right are *x* = -35 mm, -20 mm, -5 mm, 10 mm, 25 mm, and 40 mm, respectively. Blue-green clusters show the significant decreased NII-RF in PI-ME/CFS participants. The colour bar represents 1-p values, with higher values indicating greater levels of statistical significance.


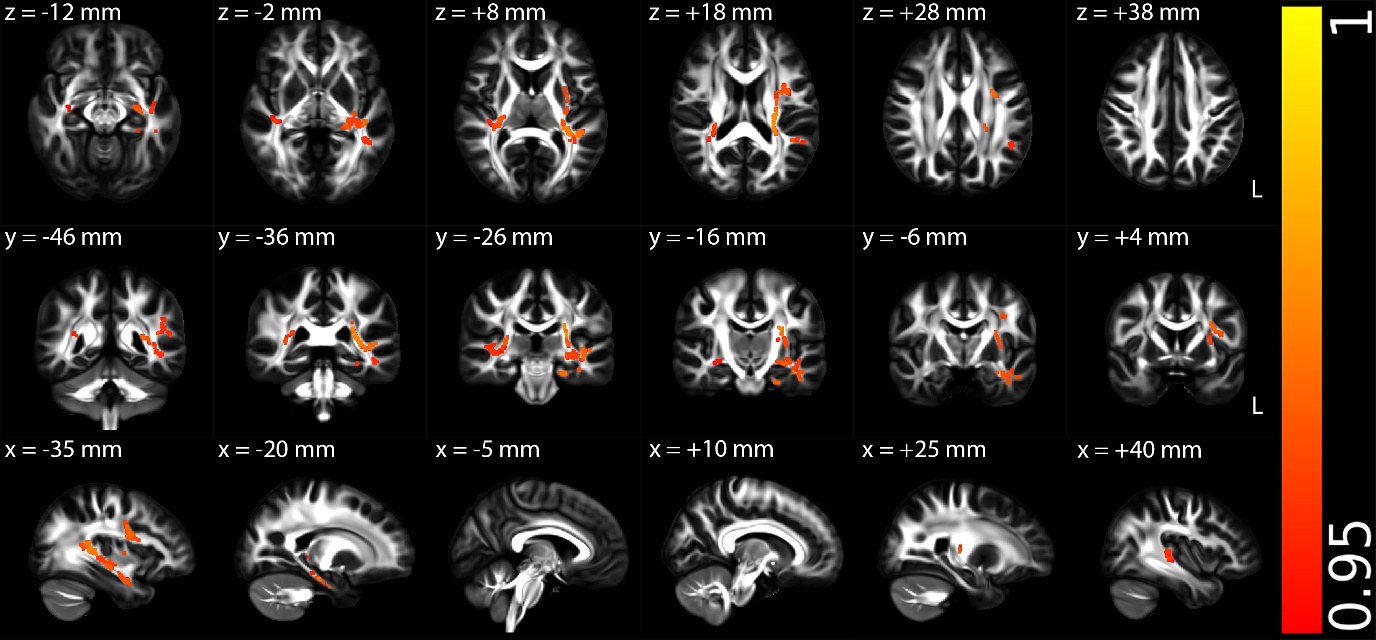


Supplementary Fig. S2: The tract-based spatial statistics (TBSS) results of diffusion-based neuroinflammation imaging-derived fibre fraction (NII-FF) between the healthy control (n = 58) and post-infectious ME/CFS (PI-ME/CFS) (n = 43) participant groups. Results are displayed in Montreal Neurological Institute (MNI) 152 standard space based on the reference FSL_HCP1065 fractional anisotropy 1x1x1mm standard-space image. The top row shows the results from six different axial slices, where the *z*-coordinates in MNI space from left to right are *z* = -12 mm, -2 mm, 8 mm, 18 mm, 28 mm, and 38 mm, respectively. The middle row shows the results from six different coronal slices, where the *y*-coordinates in MNI space from left to right are *y* = -46 mm, -36 mm, -26 mm, -16 mm, -6 mm, and 4 mm, respectively. The bottom row shows the results from six different sagittal slices, where the *x*-coordinates in MNI space from left to right are *x* = -35 mm, -20 mm, -5 mm, 10 mm, 25 mm, and 40 mm, respectively. Red-yellow clusters show the significant increased NII-FF in PI-ME/CFS participants. The colour bar represents 1-p values, with higher values indicating greater levels of statistical significance.


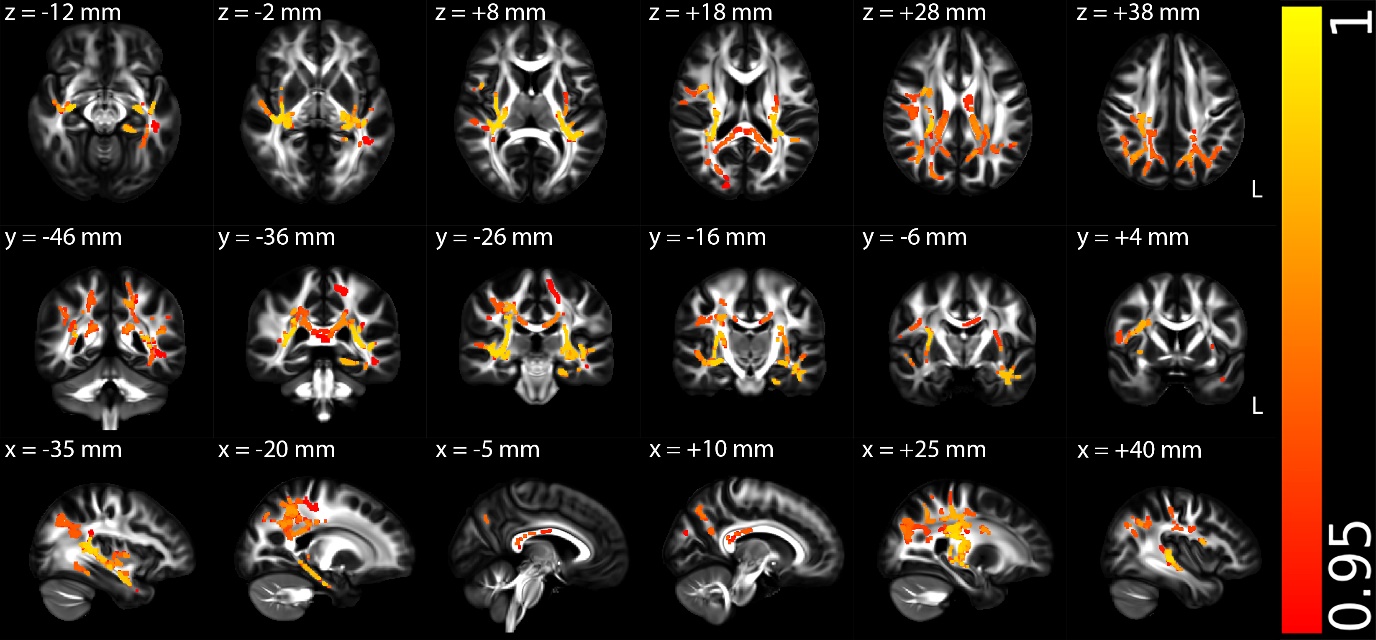


Supplementary Fig. S3: The tract-based spatial statistics (TBSS) results of diffusion-based neuroinflammation imaging-derived axial diffusivity (NII-AD) between the healthy control (n = 58) and post-infectious ME/CFS (PI-ME/CFS) (n = 43) participant groups. Results are displayed in Montreal Neurological Institute (MNI) 152 standard space based on the reference FSL_HCP1065 fractional anisotropy 1x1x1mm standard-space image. The top row shows the results from six different axial slices, where the *z*-coordinates in MNI space from left to right are *z* = -12 mm, -2 mm, 8 mm, 18 mm, 28 mm, and 38 mm, respectively. The middle row shows the results from six different coronal slices, where the *y*-coordinates in MNI space from left to right are *y* = -46 mm, -36 mm, -26 mm, -16 mm, -6 mm, and 4 mm, respectively. The bottom row shows the results from six different sagittal slices, where the *x*-coordinates in MNI space from left to right are *x* = -35 mm, -20 mm, -5 mm, 10 mm, 25 mm, and 40 mm, respectively. Red-yellow clusters show the significant increased NII-AD in PI-ME/CFS participants. The colour bar represents 1-p values, with higher values indicating greater levels of statistical significance.


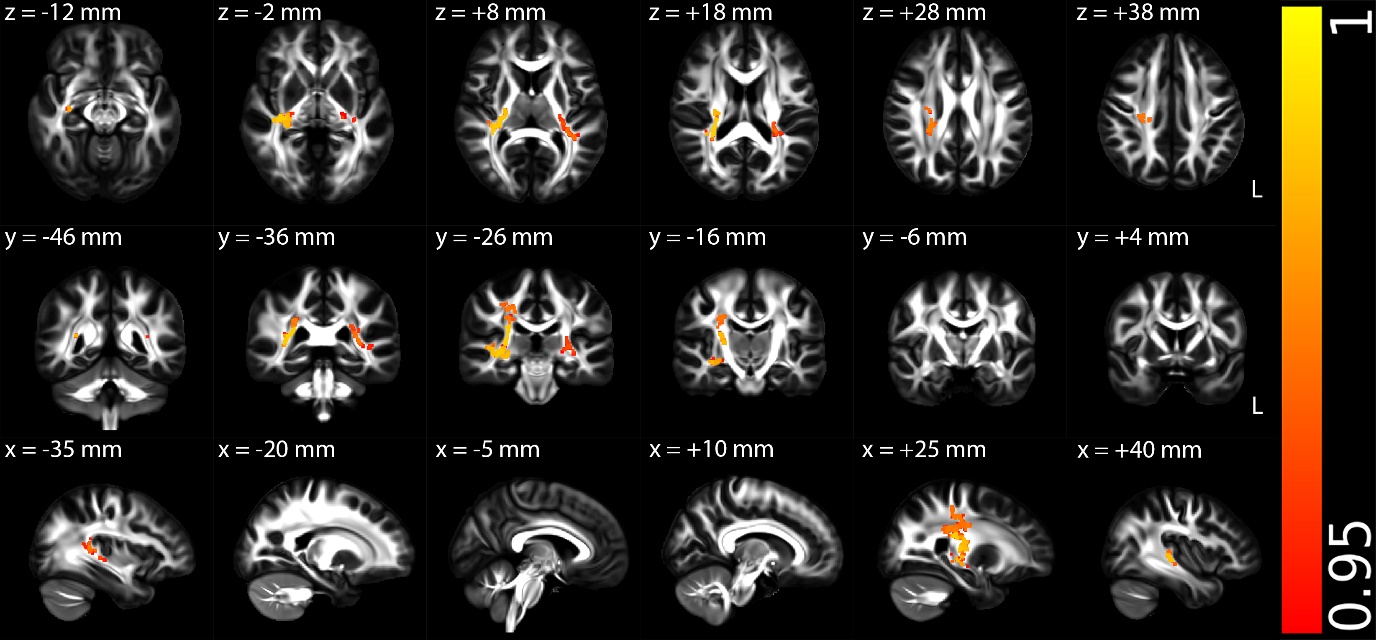


Supplementary Fig. S4: The tract-based spatial statistics (TBSS) results of diffusion-based neuroinflammation imaging-derived fractional anisotropy (NII-FA) between the healthy control (n = 58) and post-infectious ME/CFS (PI-ME/CFS) (n = 43) participant groups. Results are displayed in Montreal Neurological Institute (MNI) 152 standard space based on the reference FSL_HCP1065 fractional anisotropy 1x1x1mm standard-space image. The top row shows the results from six different axial slices, where the *z*-coordinates in MNI space from left to right are *z* = -12 mm, -2 mm, 8 mm, 18 mm, 28 mm, and 38 mm, respectively. The middle row shows the results from six different coronal slices, where the *y*-coordinates in MNI space from left to right are *y* = -46 mm, -36 mm, -26 mm, -16 mm, -6 mm, and 4 mm, respectively. The bottom row shows the results from six different sagittal slices, where the *x*-coordinates in MNI space from left to right are *x* = -35 mm, -20 mm, -5 mm, 10 mm, 25 mm, and 40 mm, respectively. Red-yellow clusters show the significant increased NII-FA in PI-ME/CFS participants. The colour bar represents 1-p values, with higher values indicating greater levels of statistical significance.


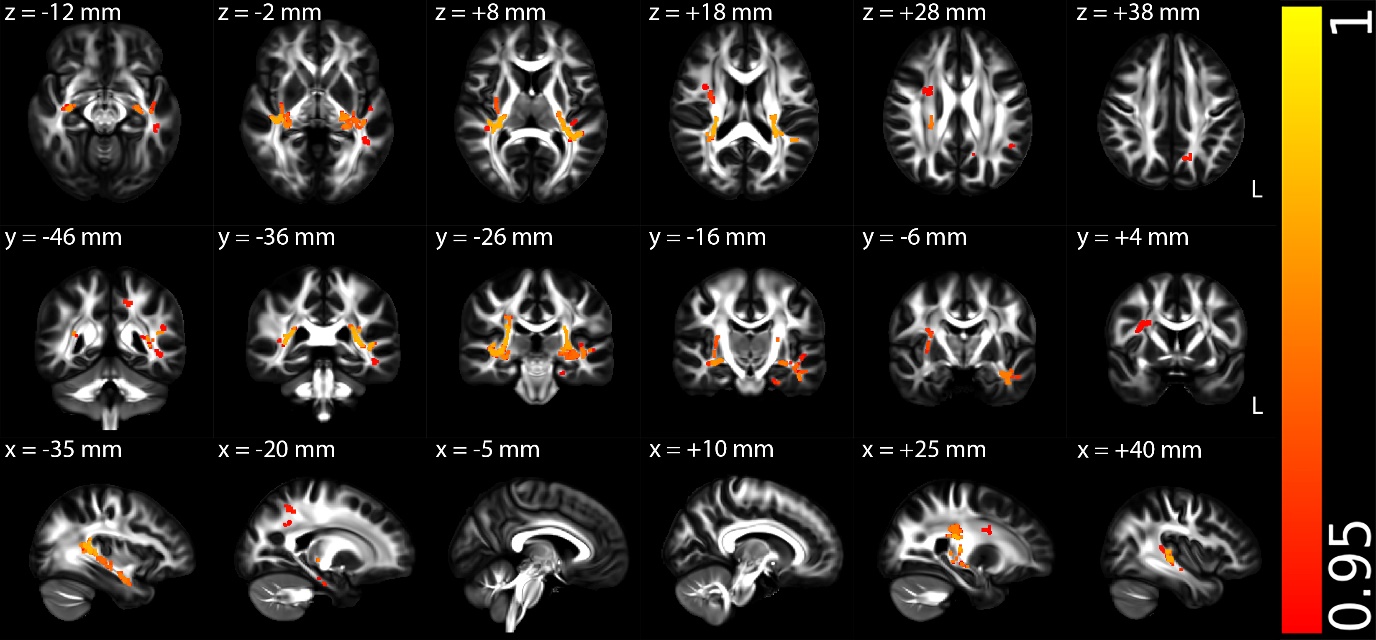


Supplementary Fig. S5: The tract-based spatial statistics (TBSS) results of diffusion-based neuroinflammation imaging-derived mean diffusivity (NII-MD) between the healthy control (n = 58) and post-infectious ME/CFS (PI-ME/CFS) (n = 43) participant groups. Results are displayed in Montreal Neurological Institute (MNI) 152 standard space based on the reference FSL_HCP1065 fractional anisotropy 1x1x1mm standard-space image. The top row shows the results from six different axial slices, where the *z*-coordinates in MNI space from left to right are *z* = -12 mm, -2 mm, 8 mm, 18 mm, 28 mm, and 38 mm, respectively. The middle row shows the results from six different coronal slices, where the *y*-coordinates in MNI space from left to right are *y* = -46 mm, -36 mm, -26 mm, -16 mm, -6 mm, and 4 mm, respectively. The bottom row shows the results from six different sagittal slices, where the *x*-coordinates in MNI space from left to right are *x* = -35 mm, -20 mm, -5 mm, 10 mm, 25 mm, and 40 mm, respectively. Red-yellow clusters show the significant increased NII-MD in PI-ME/CFS participants. The colour bar represents 1-p values, with higher values indicating greater levels of statistical significance.


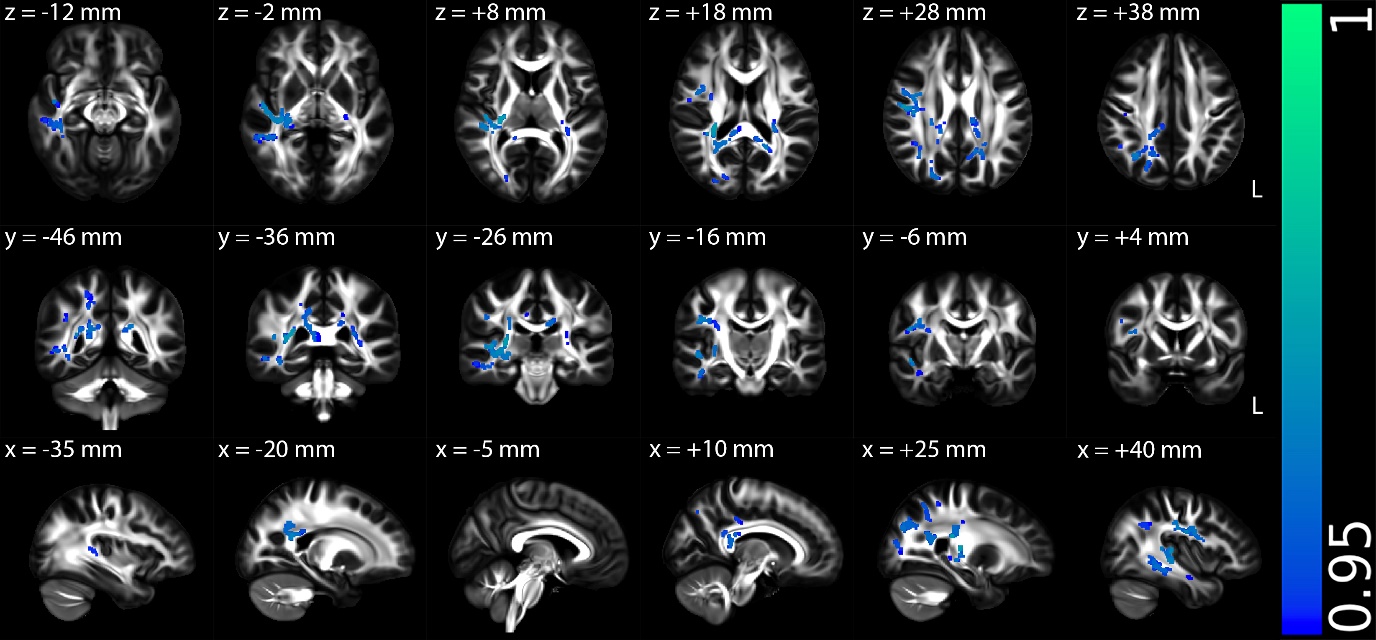


Supplementary Fig. S6: The tract-based spatial statistics (TBSS) results of diffusion-based neuroinflammation imaging-derived hindered water ratio (NII-HR) between the healthy control (n = 58) and gradual onset ME/CFS (GO-ME/CFS) (n = 33) participant groups. Results are displayed in Montreal Neurological Institute (MNI) 152 standard space based on the reference FSL_HCP1065 fractional anisotropy 1x1x1mm standard-space image. The top row shows the results from six different axial slices, where the *z*-coordinates in MNI space from left to right are *z* = -12 mm, -2 mm, 8 mm, 18 mm, 28 mm, and 38 mm, respectively. The middle row shows the results from six different coronal slices, where the *y*-coordinates in MNI space from left to right are *y* = -46 mm, -36 mm, -26 mm, -16 mm, -6 mm, and 4 mm, respectively. The bottom row shows the results from six different sagittal slices, where the *x*-coordinates in MNI space from left to right are *x* = -35 mm, -20 mm, -5 mm, 10 mm, 25 mm, and 40 mm, respectively. Blue-green clusters show the significant decreased NII-HR in GO-ME/CFS participants. The colour bar represents 1-p values, with higher values indicating greater levels of statistical significance.


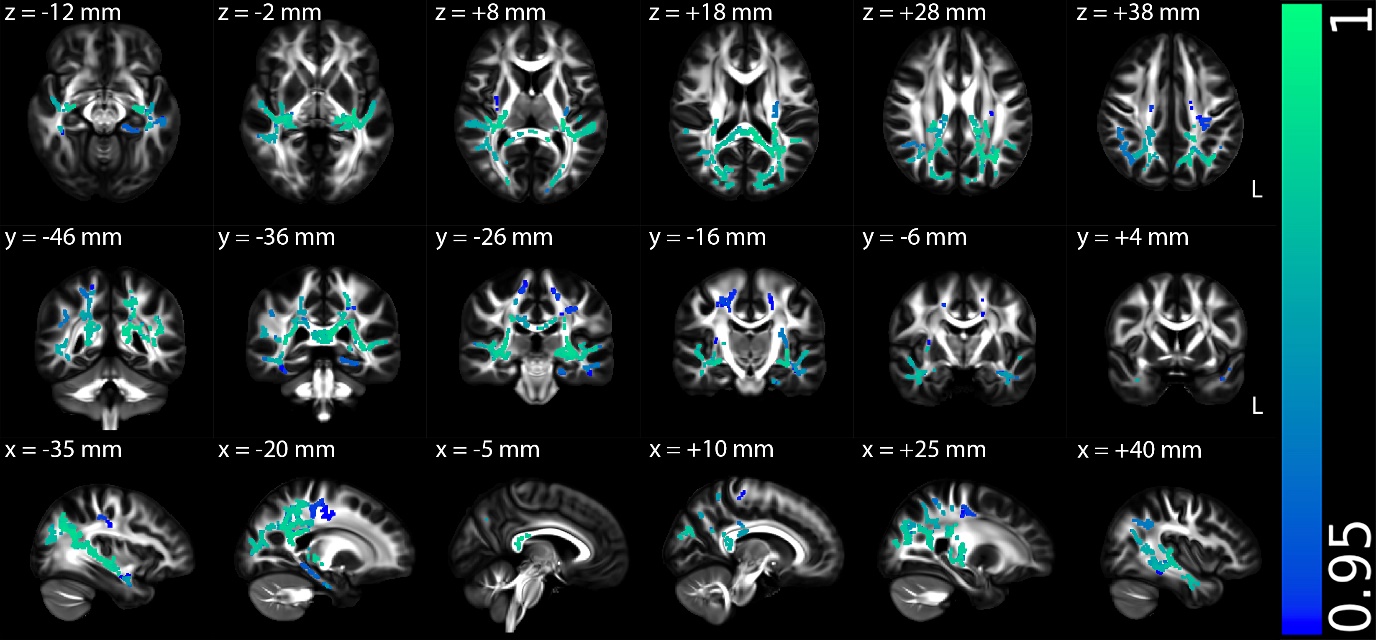


Supplementary Fig. S7: The tract-based spatial statistics (TBSS) results of diffusion-based neuroinflammation imaging-derived hindered fraction of restricted isotropic diffusion (NII-RF) between the healthy control (n = 58) and gradual onset ME/CFS (GO-ME/CFS) (n = 33) participant groups. Results are displayed in Montreal Neurological Institute (MNI) 152 standard space based on the reference FSL_HCP1065 fractional anisotropy 1x1x1mm standard-space image. The top row shows the results from six different axial slices, where the *z*-coordinates in MNI space from left to right are *z* = -12 mm, -2 mm, 8 mm, 18 mm, 28 mm, and 38 mm, respectively. The middle row shows the results from six different coronal slices, where the *y*-coordinates in MNI space from left to right are *y* = -46 mm, -36 mm, -26 mm, -16 mm, -6 mm, and 4 mm, respectively. The bottom row shows the results from six different sagittal slices, where the *x*-coordinates in MNI space from left to right are *x* = -35 mm, -20 mm, -5 mm, 10 mm, 25 mm, and 40 mm, respectively. Blue-green clusters show the significant decreased NII-RF in GO-ME/CFS participants. The colour bar represents 1-p values, with higher values indicating greater levels of statistical significance.


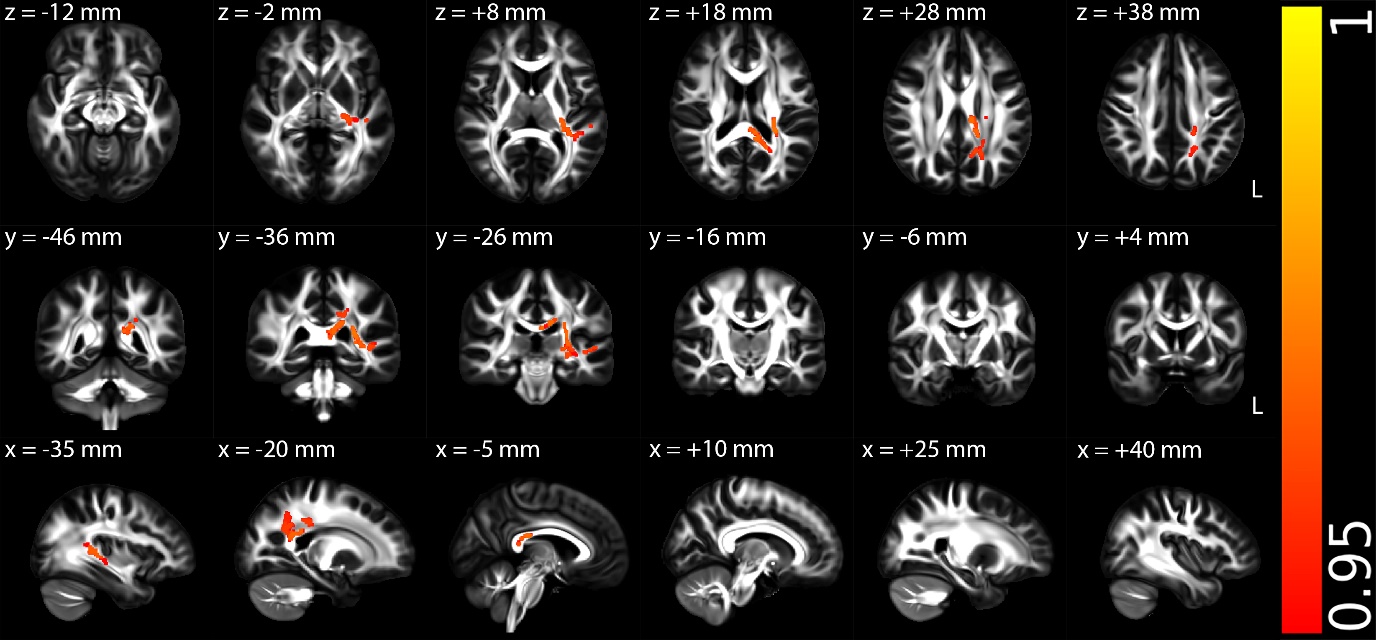


Supplementary Fig. S8: The tract-based spatial statistics (TBSS) results of diffusion-based neuroinflammation imaging-derived fibre fraction (NII-FF) between the healthy control (n = 58) and gradual onset ME/CFS (GO-ME/CFS) (n = 33) participant groups. Results are displayed in Montreal Neurological Institute (MNI) 152 standard space based on the reference FSL_HCP1065 fractional anisotropy 1x1x1mm standard-space image. The top row shows the results from six different axial slices, where the *z*-coordinates in MNI space from left to right are *z* = -12 mm, -2 mm, 8 mm, 18 mm, 28 mm, and 38 mm, respectively. The middle row shows the results from six different coronal slices, where the *y*-coordinates in MNI space from left to right are *y* = -46 mm, -36 mm, -26 mm, -16 mm, -6 mm, and 4 mm, respectively. The bottom row shows the results from six different sagittal slices, where the *x*-coordinates in MNI space from left to right are *x* = -35 mm, -20 mm, -5 mm, 10 mm, 25 mm, and 40 mm, respectively. Red-yellow clusters show the significant increased NII-FF in GO-ME/CFS participants. The colour bar represents 1-p values, with higher values indicating greater levels of statistical significance.


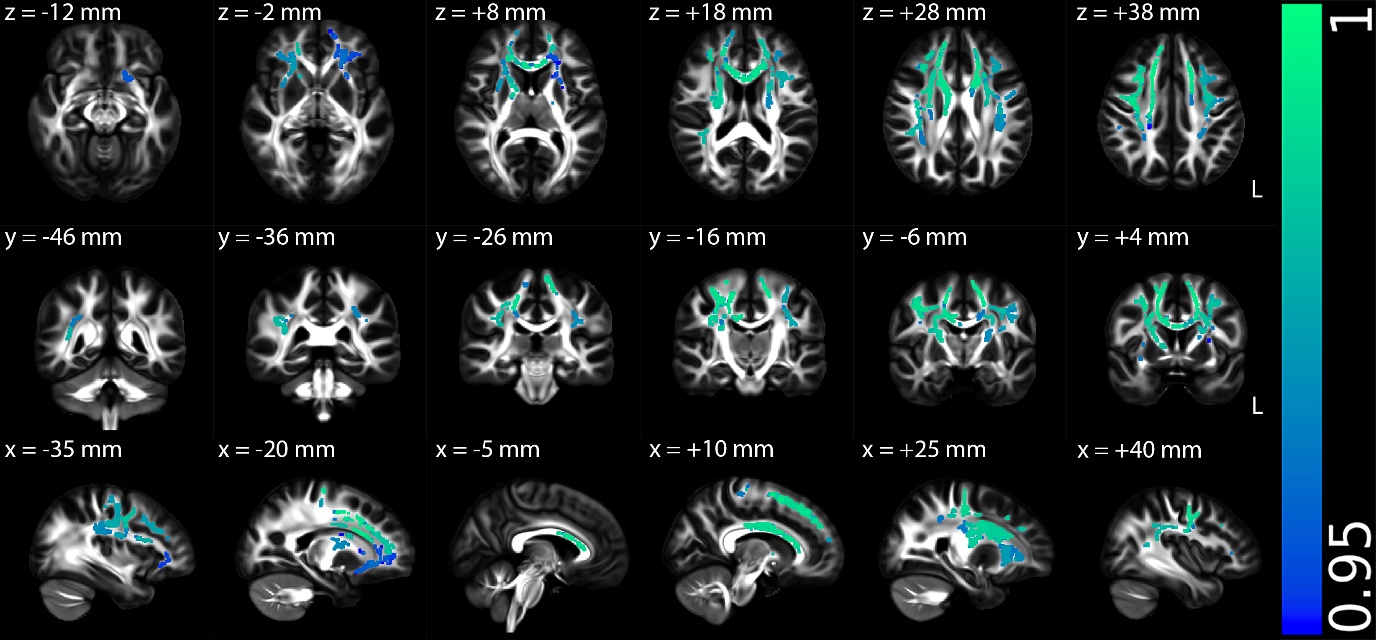


Supplementary Fig. S9: The tract-based spatial statistics (TBSS) results of diffusion-based neuroinflammation imaging-derived mean diffusivity (NII-MD) between the healthy control (n = 58) and gradual onset ME/CFS (GO-ME/CFS) (n = 33) participant groups. Results are displayed in Montreal Neurological Institute (MNI) 152 standard space based on the reference FSL_HCP1065 fractional anisotropy 1x1x1mm standard-space image. The top row shows the results from six different axial slices, where the *z*-coordinates in MNI space from left to right are *z* = -12 mm, -2 mm, 8 mm, 18 mm, 28 mm, and 38 mm, respectively. The middle row shows the results from six different coronal slices, where the *y*-coordinates in MNI space from left to right are *y* = -46 mm, -36 mm, -26 mm, -16 mm, -6 mm, and 4 mm, respectively. The bottom row shows the results from six different sagittal slices, where the *x*-coordinates in MNI space from left to right are *x* = -35 mm, -20 mm, -5 mm, 10 mm, 25 mm, and 40 mm, respectively. Blue-green clusters show the significant decreased NII-MD in GO-ME/CFS participants. The colour bar represents 1-p values, with higher values indicating greater levels of statistical significance.


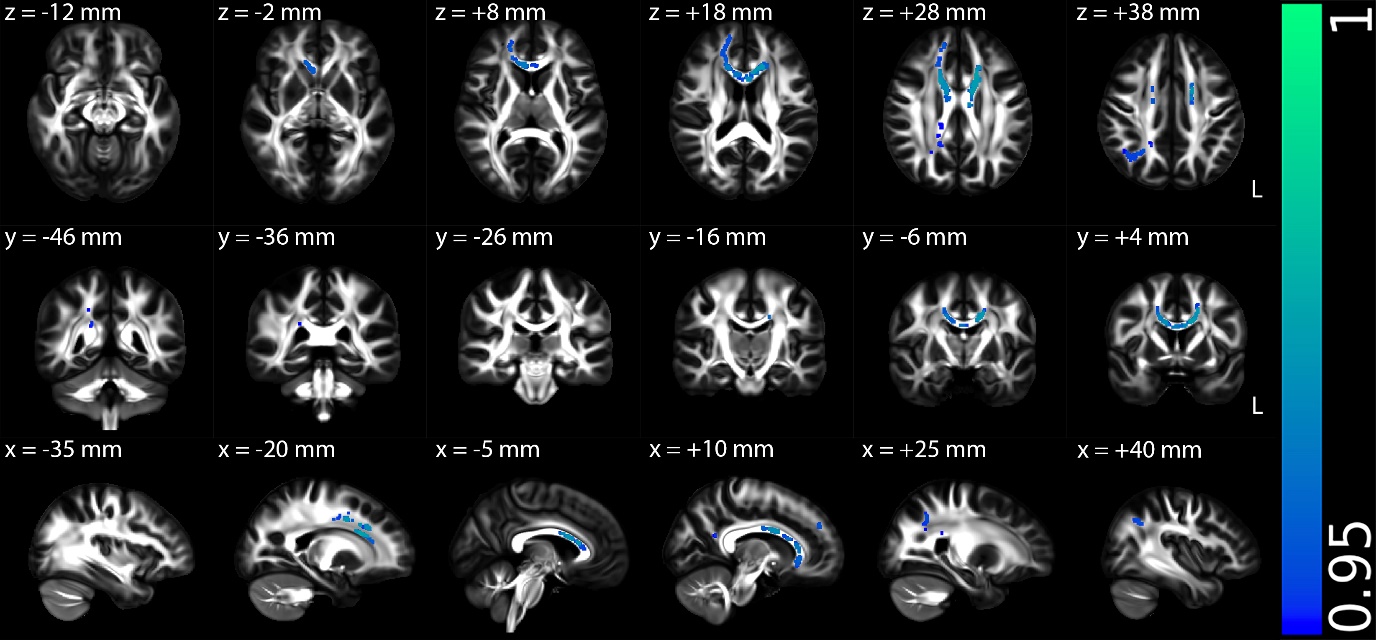


Supplementary Fig. S10: The tract-based spatial statistics (TBSS) results of diffusion-based neuroinflammation imaging-derived radial diffusivity (NII-RD) between the healthy control (n = 58) and gradual onset ME/CFS (GO-ME/CFS) (n = 33) participant groups. Results are displayed in Montreal Neurological Institute (MNI) 152 standard space based on the reference FSL_HCP1065 fractional anisotropy 1x1x1mm standard-space image. The top row shows the results from six different axial slices, where the *z*-coordinates in MNI space from left to right are *z* = -12 mm, -2 mm, 8 mm, 18 mm, 28 mm, and 38 mm, respectively. The middle row shows the results from six different coronal slices, where the *y*-coordinates in MNI space from left to right are *y* = -46 mm, -36 mm, -26 mm, -16 mm, -6 mm, and 4 mm, respectively. The bottom row shows the results from six different sagittal slices, where the *x*-coordinates in MNI space from left to right are *x* = -35 mm, -20 mm, -5 mm, 10 mm, 25 mm, and 40 mm, respectively. Blue-green clusters show the significant decreased NII-RD in GO-ME/CFS participants. The colour bar represents 1-p values, with higher values indicating greater levels of statistical significance.


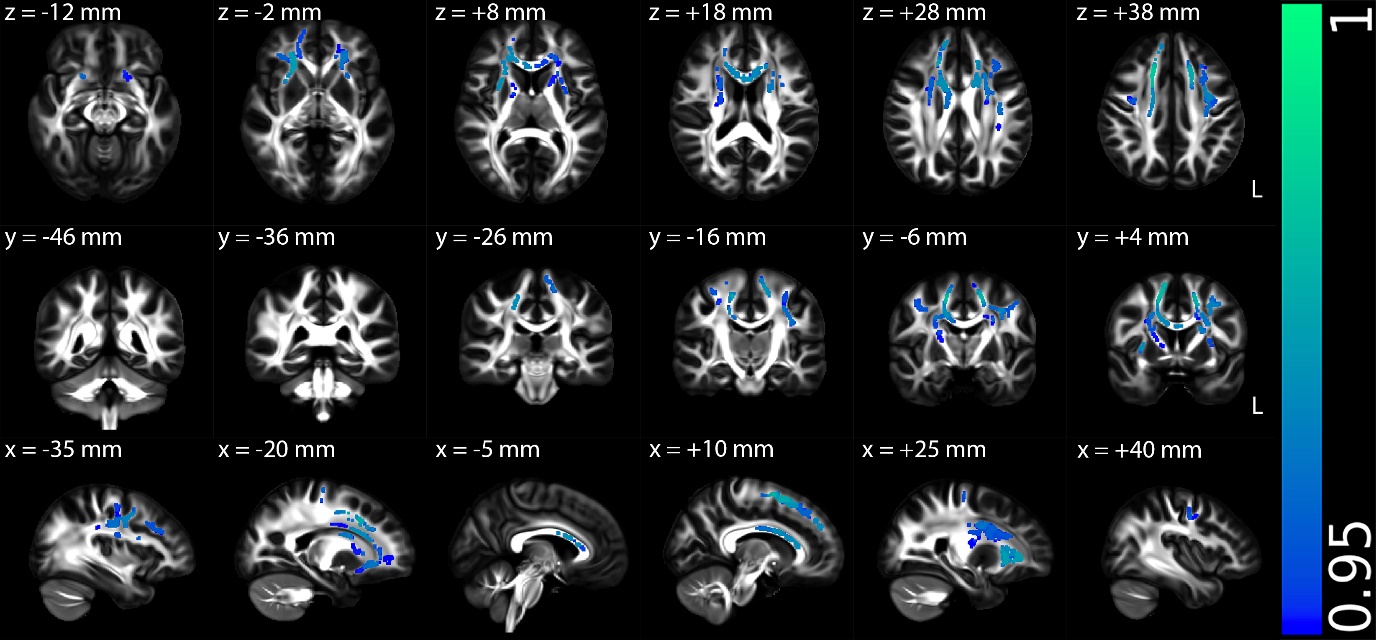


Supplementary Fig. S11: The tract-based spatial statistics (TBSS) results of diffusion-based neuroinflammation imaging-derived axial diffusivity (NII-AD) between the healthy control (n = 58) and gradual onset ME/CFS (GO-ME/CFS) (n = 33) participant groups. Results are displayed in Montreal Neurological Institute (MNI) 152 standard space based on the reference FSL_HCP1065 fractional anisotropy 1x1x1mm standard-space image. The top row shows the results from six different axial slices, where the *z*-coordinates in MNI space from left to right are *z* = -12 mm, -2 mm, 8 mm, 18 mm, 28 mm, and 38 mm, respectively. The middle row shows the results from six different coronal slices, where the *y*-coordinates in MNI space from left to right are *y* = -46 mm, -36 mm, -26 mm, -16 mm, -6 mm, and 4 mm, respectively. The bottom row shows the results from six different sagittal slices, where the *x*-coordinates in MNI space from left to right are *x* = -35 mm, -20 mm, -5 mm, 10 mm, 25 mm, and 40 mm, respectively. Blue-green clusters show the significant decreased NII-AD in GO-ME/CFS participants. The colour bar represents 1-p values, with higher values indicating greater levels of statistical significance.


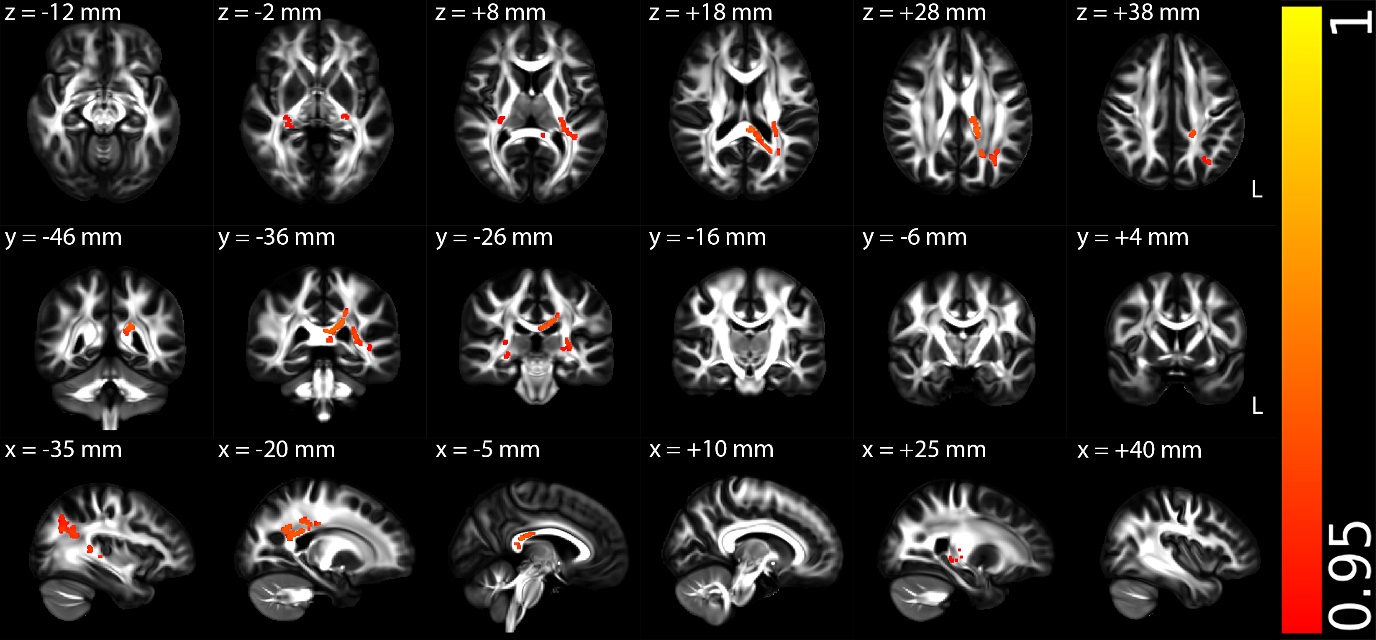


Supplementary Fig. S12: The tract-based spatial statistics (TBSS) results of diffusion-based neuroinflammation imaging-derived axial diffusivity (NII-AD) between the control (n = 58) and gradual onset ME/CFS (GO-ME/CFS) (n = 33) participant groups. Results are displayed in Montreal Neurological Institute (MNI) 152 standard space based on the reference FSL_HCP1065 fractional anisotropy 1x1x1mm standard-space image. The top row shows the results from six different axial slices, where the *z*-coordinates in MNI space from left to right are *z* = -12 mm, -2 mm, 8 mm, 18 mm, 28 mm, and 38 mm, respectively. The middle row shows the results from six different coronal slices, where the *y*-coordinates in MNI space from left to right are *y* = -46 mm, -36 mm, -26 mm, -16 mm, -6 mm, and 4 mm, respectively. The bottom row shows the results from six different sagittal slices, where the *x*-coordinates in MNI space from left to right are *x* = -35 mm, -20 mm, -5 mm, 10 mm, 25 mm, and 40 mm, respectively. Red-yellow clusters show the significant increased NII-AD in GO-ME/CFS participants. The colour bar represents 1-p values, with higher values indicating greater levels of statistical significance.


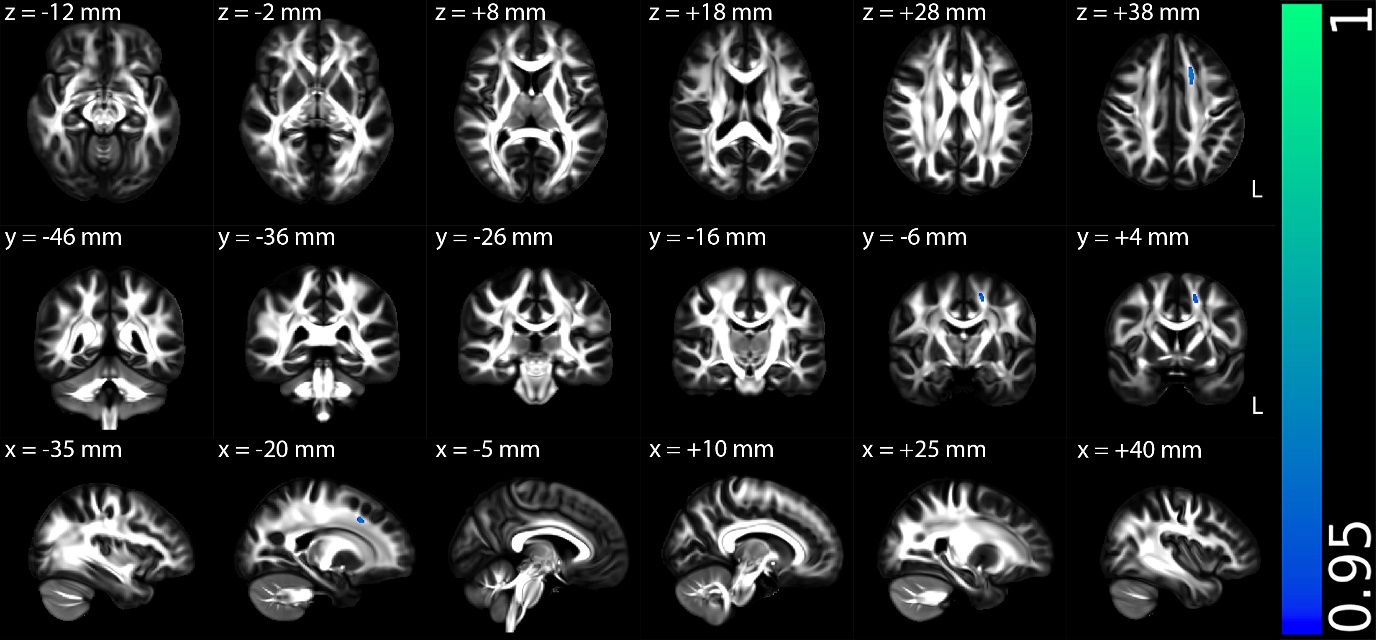


Supplementary Fig. S13: The tract-based spatial statistics (TBSS) results of diffusion-based neuroinflammation imaging-derived fractional anisotropy (NII-FA) between the healthy control (n = 58) and gradual onset ME/CFS (GO-ME/CFS) (n = 33) participant groups. Results are displayed in Montreal Neurological Institute (MNI) 152 standard space based on the reference FSL_HCP1065 fractional anisotropy 1x1x1mm standard-space image. The top row shows the results from six different axial slices, where the *z*-coordinates in MNI space from left to right are *z* = -12 mm, -2 mm, 8 mm, 18 mm, 28 mm, and 38 mm, respectively. The middle row shows the results from six different coronal slices, where the *y*-coordinates in MNI space from left to right are *y* = -46 mm, -36 mm, -26 mm, -16 mm, -6 mm, and 4 mm, respectively. The bottom row shows the results from six different sagittal slices, where the *x*-coordinates in MNI space from left to right are *x* = -35 mm, -20 mm, -5 mm, 10 mm, 25 mm, and 40 mm, respectively. Blue-green clusters show the significant decreased NII-FA in GO-ME/CFS participants. The colour bar represents 1-p values, with higher values indicating greater levels of statistical significance.


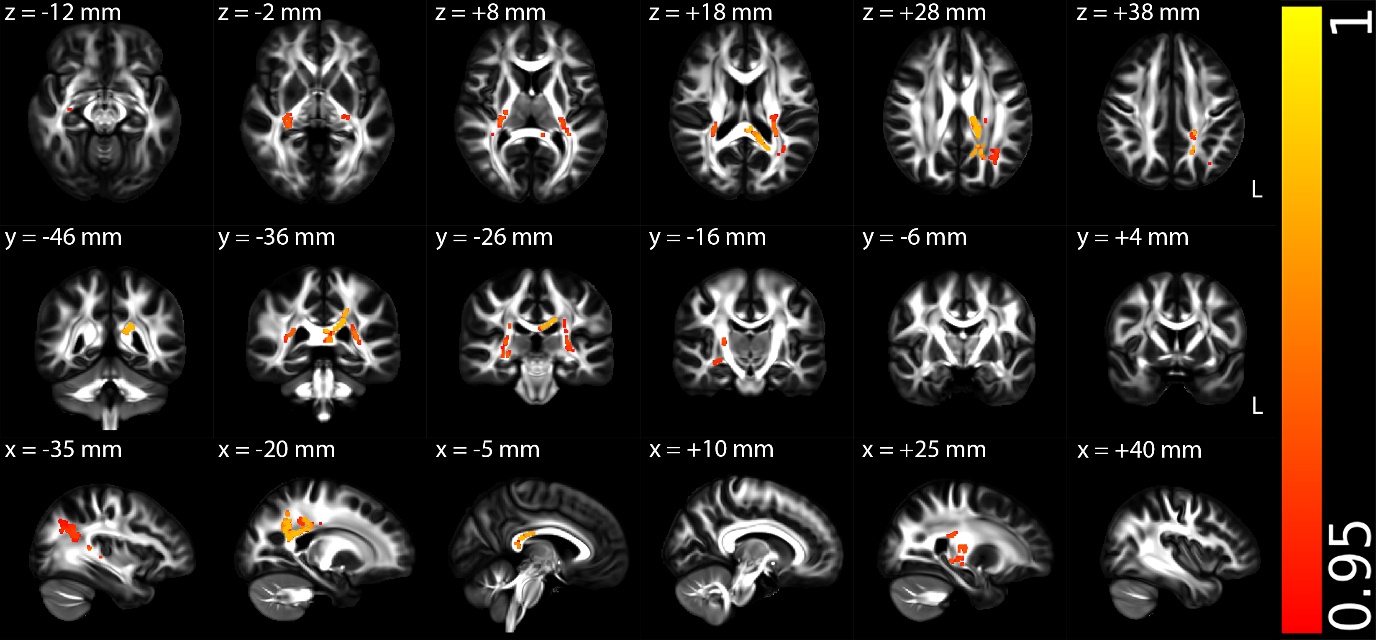


Supplementary Fig. S14: The tract-based spatial statistics (TBSS) results of diffusion-based neuroinflammation imaging-derived fractional anisotropy (NII-FA) between the healthy control (n = 58) and gradual onset ME/CFS (GO-ME/CFS) (n = 33) participant groups. Results are displayed in Montreal Neurological Institute (MNI) 152 standard space based on the reference FSL_HCP1065 fractional anisotropy 1x1x1mm standard-space image. The top row shows the results from six different axial slices, where the *z*-coordinates in MNI space from left to right are *z* = -12 mm, -2 mm, 8 mm, 18 mm, 28 mm, and 38 mm, respectively. The middle row shows the results from six different coronal slices, where the *y*-coordinates in MNI space from left to right are *y* = -46 mm, -36 mm, -26 mm, -16 mm, -6 mm, and 4 mm, respectively. The bottom row shows the results from six different sagittal slices, where the *x*-coordinates in MNI space from left to right are *x* = -35 mm, -20 mm, -5 mm, 10 mm, 25 mm, and 40 mm, respectively. Red-yellow clusters show the significant increased NII-FA in GO-ME/CFS participants. The colour bar represents 1-p values, with higher values indicating greater levels of statistical significance.


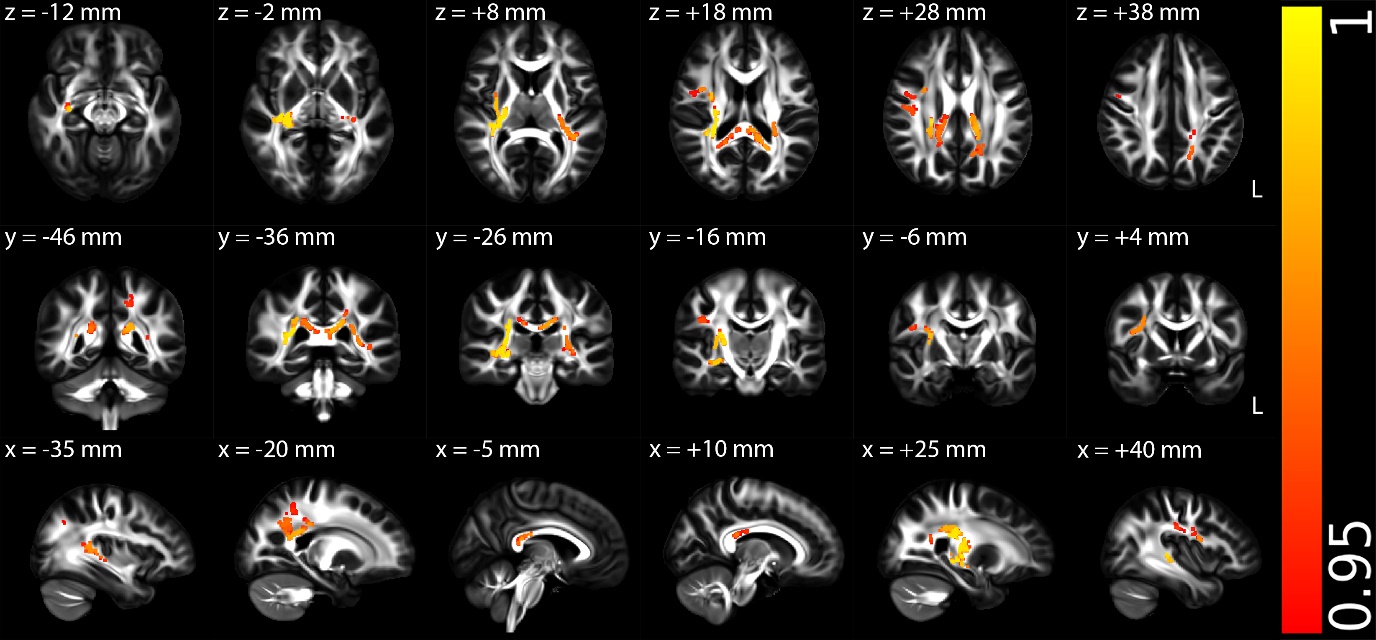


Supplementary Fig. S15: Tract-based spatial statistics (TBSS) results comparing diffusion-based neuroinflammation imaging-derived fractional anisotropy (NII-FA) between the healthy control (n = 67) and ME/CFS (n = 67) participant groups. Results are displayed in Montreal Neurological Institute (MNI) 152 standard space based on the reference FSL_HCP1065 fractional anisotropy 1x1x1mm standard-space image. The top row shows the results from six different axial slices, where the *z*-coordinates in MNI space from left to right are *z* = -12 mm, -2 mm, 8 mm, 18 mm, 28 mm, and 38 mm, respectively. The middle row shows the results from six different coronal slices, where the *y*-coordinates in MNI space from left to right are *y* = -46 mm, -36 mm, -26 mm, -16 mm, -6 mm, and 4 mm, respectively. The bottom row shows the results from six different sagittal slices, where the *x*-coordinates in MNI space from left to right are *x* = -35 mm, -20 mm, -5 mm, 10 mm, 25 mm, and 40 mm, respectively. Red-yellow clusters show the significant increased NII-FA in ME/CFS participants. The colour bar represents 1-p values (a dimensionless probability measure), with higher values indicating greater levels of statistical significance.


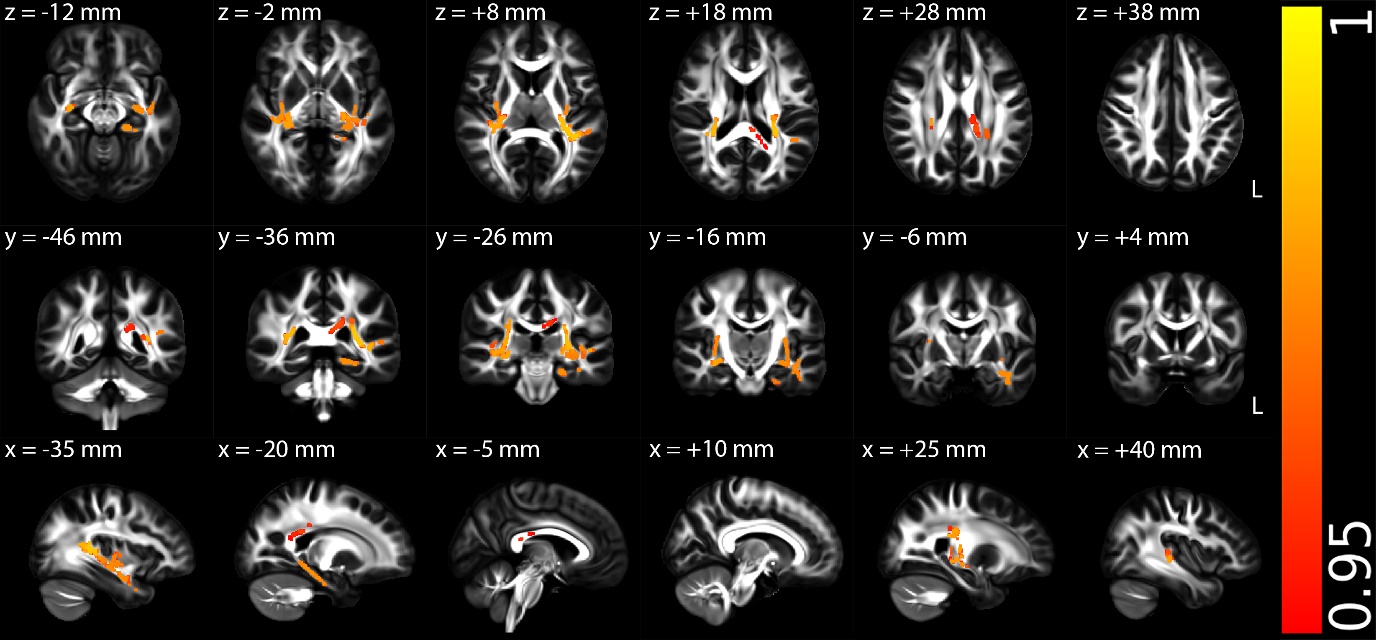


Supplementary Fig. S16: Tract-based spatial statistics (TBSS) results comparing diffusion-based neuroinflammation imaging-derived axial diffusivity (NII-AD) between the healthy control (n = 67) and ME/CFS (n = 67) participant groups. Results are displayed in Montreal Neurological Institute (MNI) 152 standard space based on the reference FSL_HCP1065 fractional anisotropy 1x1x1mm standard-space image. The top row shows the results from six different axial slices, where the *z*-coordinates in MNI space from left to right are *z* = -12 mm, -2 mm, 8 mm, 18 mm, 28 mm, and 38 mm, respectively. The middle row shows the results from six different coronal slices, where the *y*-coordinates in MNI space from left to right are *y* = -46 mm, -36 mm, -26 mm, -16 mm, -6 mm, and 4 mm, respectively. The bottom row shows the results from six different sagittal slices, where the *x*-coordinates in MNI space from left to right are *x* = -35 mm, -20 mm, -5 mm, 10 mm, 25 mm, and 40 mm, respectively. Red-yellow clusters show the significant increased NII-AD in ME/CFS participants. The colour bar represents 1-p values (a dimensionless probability measure), with higher values indicating greater levels of statistical significance.


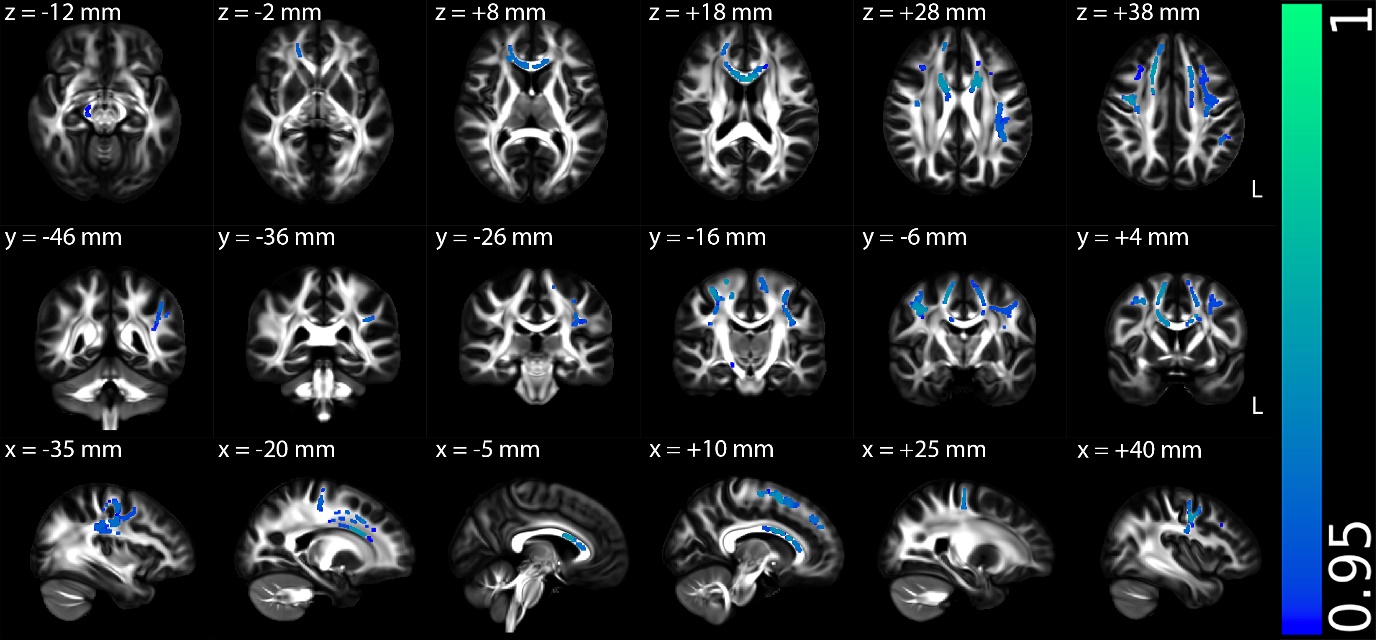


Supplementary Fig. S17: Tract-based spatial statistics (TBSS) results comparing diffusion-based neuroinflammation imaging-derived axial diffusivity (NII-AD) between the healthy control (n = 67) and ME/CFS (n = 67) participant groups. Results are displayed in Montreal Neurological Institute (MNI) 152 standard space based on the reference FSL_HCP1065 fractional anisotropy 1x1x1mm standard-space image. The top row shows the results from six different axial slices, where the *z*-coordinates in MNI space from left to right are *z* = -12 mm, -2 mm, 8 mm, 18 mm, 28 mm, and 38 mm, respectively. The middle row shows the results from six different coronal slices, where the *y*-coordinates in MNI space from left to right are *y* = -46 mm, -36 mm, -26 mm, -16 mm, -6 mm, and 4 mm, respectively. The bottom row shows the results from six different sagittal slices, where the *x*-coordinates in MNI space from left to right are *x* = -35 mm, -20 mm, -5 mm, 10 mm, 25 mm, and 40 mm, respectively. Blue-green clusters show the significant decreased NII-AD in ME/CFS participants. The colour bar represents 1-p values (a dimensionless probability measure), with higher values indicating greater levels of statistical significance.


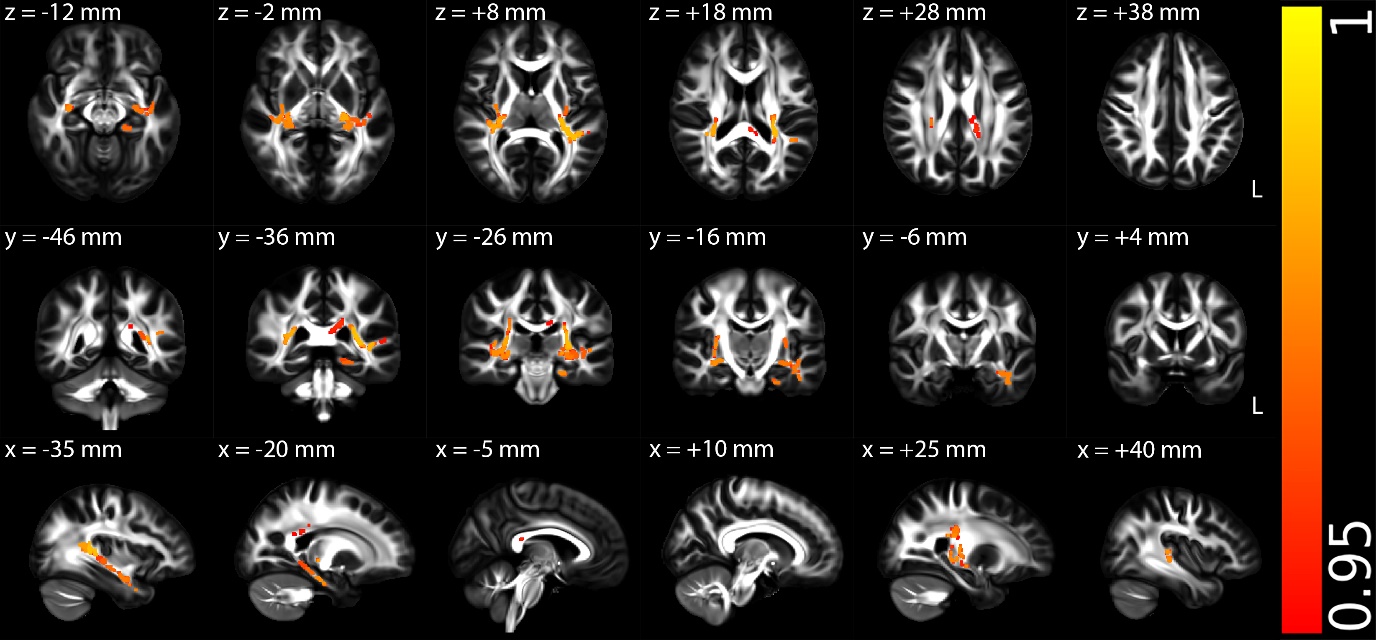


Supplementary Fig. S18: Tract-based spatial statistics (TBSS) results comparing diffusion-based neuroinflammation imaging-derived mean diffusivity (NII-MD) between the healthy control (n = 67) and ME/CFS (n = 67) participant groups. Results are displayed in Montreal Neurological Institute (MNI) 152 standard space based on the reference FSL_HCP1065 fractional anisotropy 1x1x1mm standard-space image. The top row shows the results from six different axial slices, where the *z*-coordinates in MNI space from left to right are *z* = -12 mm, -2 mm, 8 mm, 18 mm, 28 mm, and 38 mm, respectively. The middle row shows the results from six different coronal slices, where the *y*-coordinates in MNI space from left to right are *y* = -46 mm, -36 mm, -26 mm, -16 mm, -6 mm, and 4 mm, respectively. The bottom row shows the results from six different sagittal slices, where the *x*-coordinates in MNI space from left to right are *x* = -35 mm, -20 mm, -5 mm, 10 mm, 25 mm, and 40 mm, respectively. Red-yellow clusters show the significant increased NII-MD in ME/CFS participants. The colour bar represents 1-p values (a dimensionless probability measure), with higher values indicating greater levels of statistical significance.


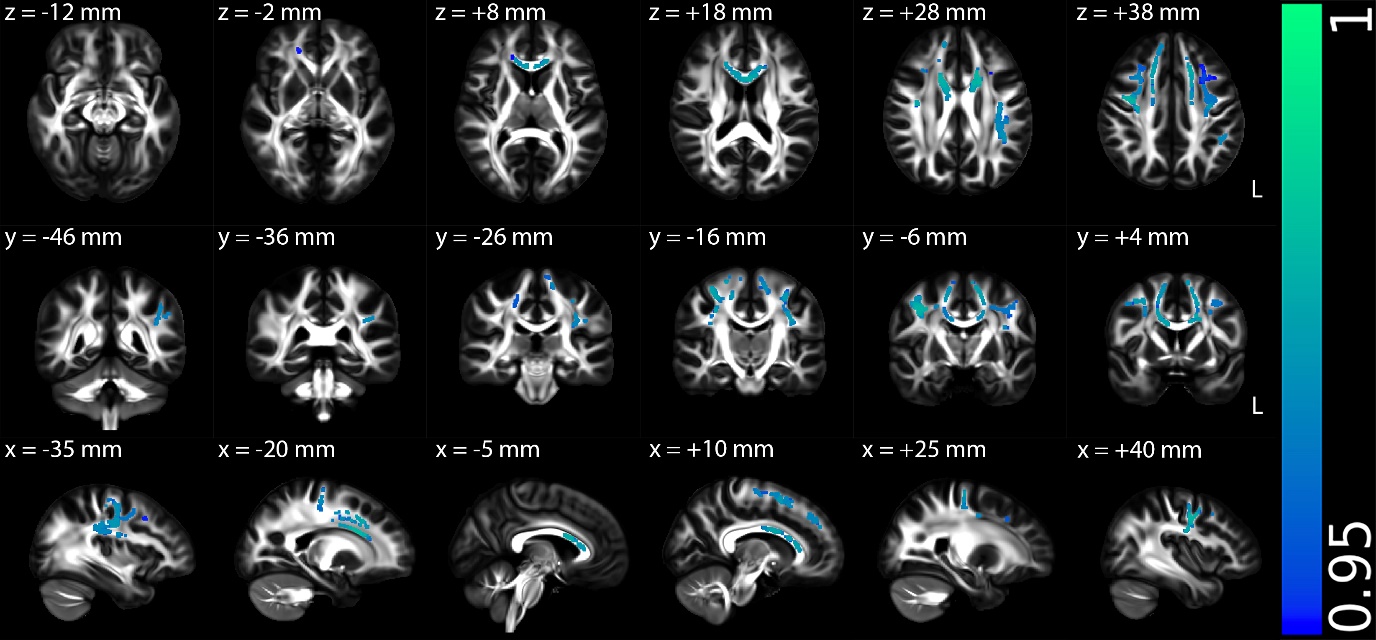


Supplementary Fig. S19: Tract-based spatial statistics (TBSS) results comparing diffusion-based neuroinflammation imaging-derived mean diffusivity (NII-MD) between the healthy control (n = 67) and ME/CFS (n = 67) participant groups. Results are displayed in Montreal Neurological Institute (MNI) 152 standard space based on the reference FSL_HCP1065 fractional anisotropy 1x1x1mm standard-space image. The top row shows the results from six different axial slices, where the *z*-coordinates in MNI space from left to right are *z* = -12 mm, -2 mm, 8 mm, 18 mm, 28 mm, and 38 mm, respectively. The middle row shows the results from six different coronal slices, where the *y*-coordinates in MNI space from left to right are *y* = -46 mm, -36 mm, -26 mm, -16 mm, -6 mm, and 4 mm, respectively. The bottom row shows the results from six different sagittal slices, where the *x*-coordinates in MNI space from left to right are *x* = -35 mm, -20 mm, -5 mm, 10 mm, 25 mm, and 40 mm, respectively. Blue-green clusters show the significant decreased NII-MD in ME/CFS participants. The colour bar represents 1-p values (a dimensionless probability measure), with higher values indicating greater levels of statistical significance.


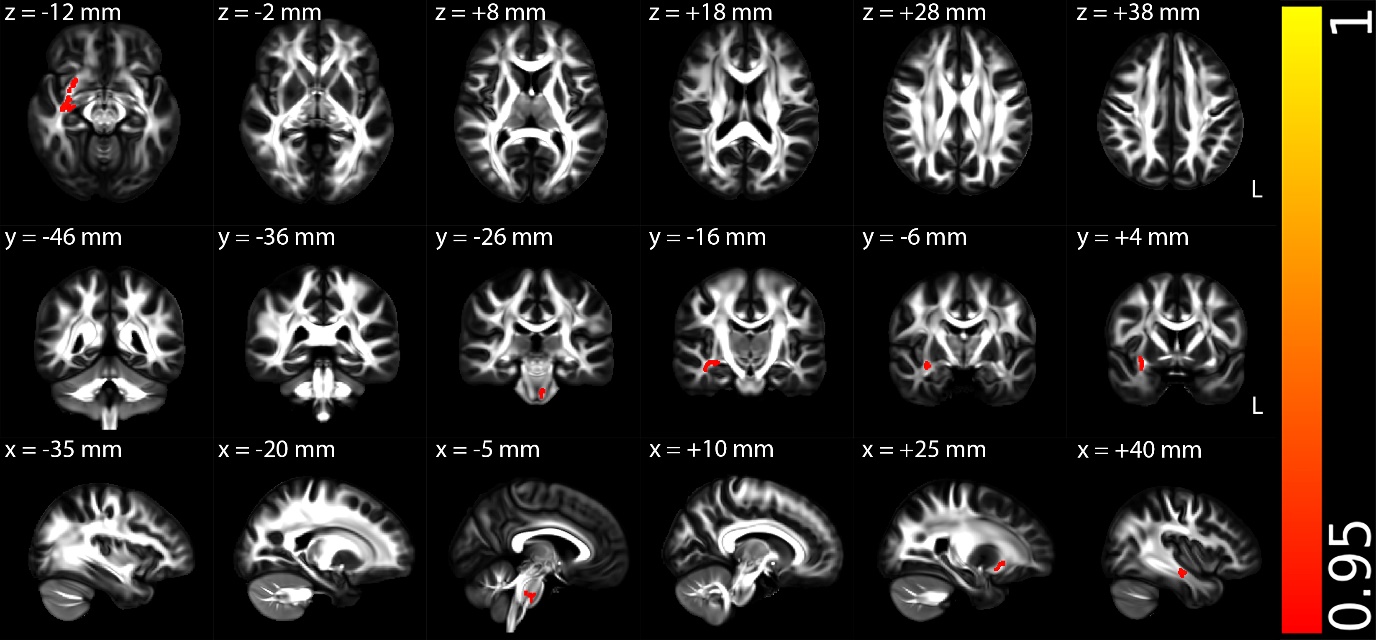


Supplementary Fig. S20: Tract-based spatial statistics (TBSS) results comparing diffusion tensor imaging-derived axial diffusivity (DTI-AD) between the healthy control (n = 67) and ME/CFS (n = 67) participant groups. Results are displayed in Montreal Neurological Institute (MNI) 152 standard space based on the reference FSL_HCP1065 fractional anisotropy 1x1x1mm standard-space image. The top row shows the results from six different axial slices, where the *z*-coordinates in MNI space from left to right are *z* = -12 mm, -2 mm, 8 mm, 18 mm, 28 mm, and 38 mm, respectively. The middle row shows the results from six different coronal slices, where the *y*-coordinates in MNI space from left to right are *y* = -46 mm, -36 mm, -26 mm, -16 mm, -6 mm, and 4 mm, respectively. The bottom row shows the results from six different sagittal slices, where the *x*-coordinates in MNI space from left to right are *x* = -35 mm, -20 mm, -5 mm, 10 mm, 25 mm, and 40 mm, respectively. Red-yellow clusters show the significant increased DTI-AD in ME/CFS participants. The colour bar represents 1-p values (a dimensionless probability measure), with higher values indicating greater levels of statistical significance.


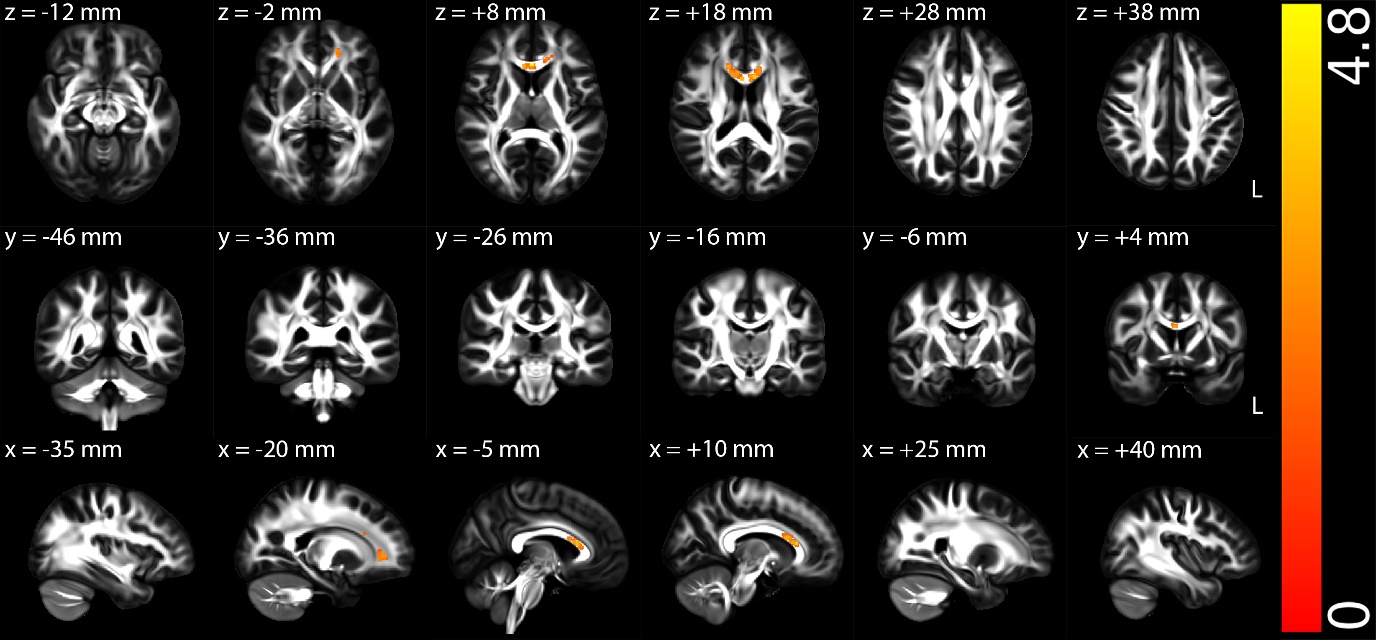


Supplementary Fig. S21: Multiple regression t-statistical results of diffusion-based neuroinflammation imaging-derived axial diffusivity (NII-AD) with mental component summary (MCS) among all participants (67 healthy controls and 67 ME/CFS participants). Results are displayed in Montreal Neurological Institute (MNI) 152 standard space based on the reference FSL_HCP1065 fractional anisotropy 1x1x1mm standard-space image. The top row shows the results from six different axial slices, where the *z*-coordinates in MNI space from left to right are *z* = -12 mm, -2 mm, 8 mm, 18 mm, 28 mm, and 38 mm, respectively. The middle row shows the results from six different coronal slices, where the *y*-coordinates in MNI space from left to right are *y* = -46 mm, -36 mm, -26 mm, -16 mm, -6 mm, and 4 mm, respectively. The bottom row shows the results from six different sagittal slices, where the *x*-coordinates in MNI space from left to right are *x* = -35 mm, -20 mm, -5 mm, 10 mm, 25 mm, and 40 mm, respectively. Red-yellow clusters show the significant positive association (FWE corrected p < 0.05 with TFCE) of NII-AD with MCS for all participants. The colour bar represents t-statistical values.


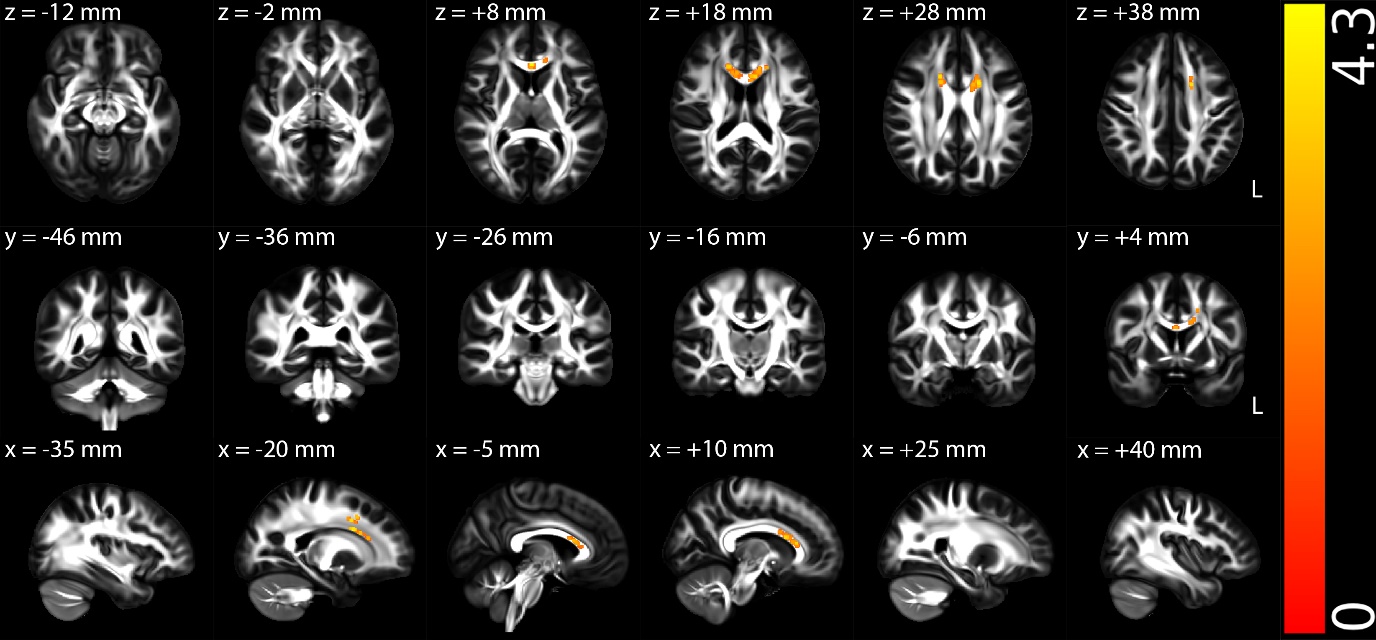


Supplementary Fig. S22: Multiple regression t-statistical results of diffusion-based neuroinflammation imaging-derived mean diffusivity (NII-MD) with mental component summary (MCS) among all participants (67 healthy controls and 67 ME/CFS participants). Results are displayed in Montreal Neurological Institute (MNI) 152 standard space based on the reference FSL_HCP1065 fractional anisotropy 1x1x1mm standard-space image. The top row shows the results from six different axial slices, where the *z*-coordinates in MNI space from left to right are *z* = -12 mm, -2 mm, 8 mm, 18 mm, 28 mm, and 38 mm, respectively. The middle row shows the results from six different coronal slices, where the *y*-coordinates in MNI space from left to right are *y* = -46 mm, -36 mm, -26 mm, -16 mm, -6 mm, and 4 mm, respectively. The bottom row shows the results from six different sagittal slices, where the *x*-coordinates in MNI space from left to right are *x* = -35 mm, -20 mm, -5 mm, 10 mm, 25 mm, and 40 mm, respectively. Red-yellow clusters show the significant positive association (FWE corrected p < 0.05 with TFCE) of NII-MD with MCS for all participants. The colour bar represents t-statistical values.


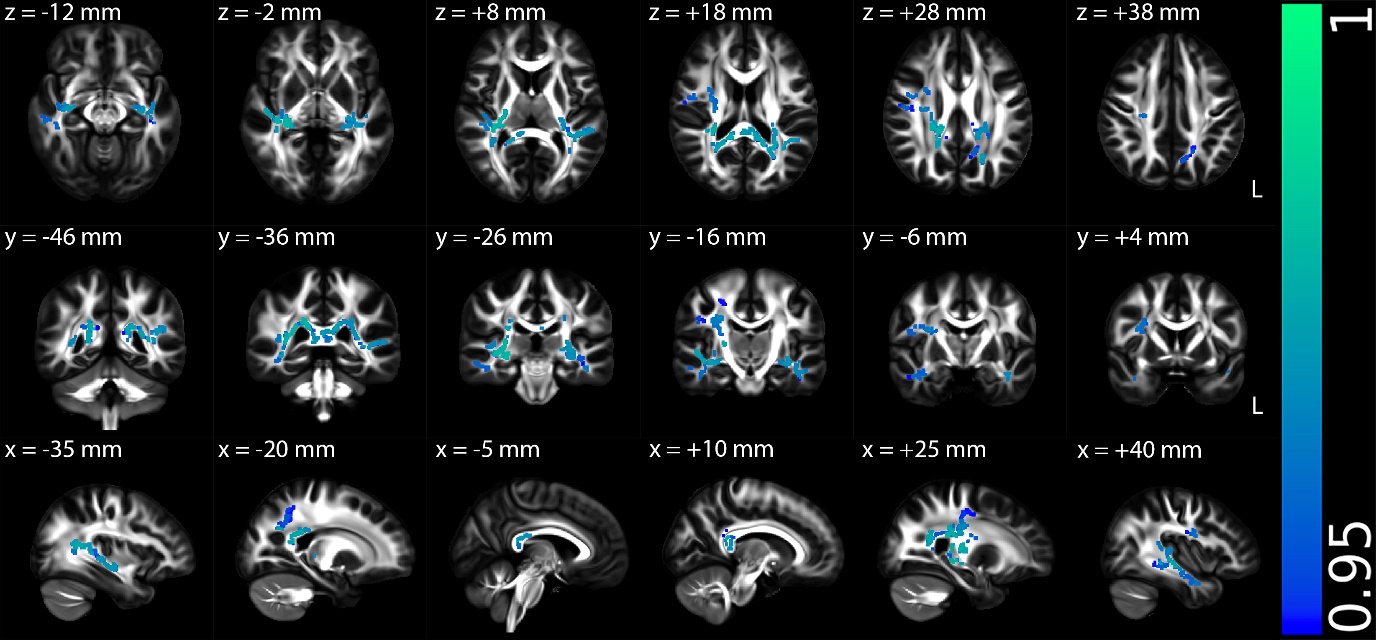


Supplementary Fig. S23: Tract-based spatial statistics (TBSS) results comparing diffusion-based neuroinflammation imaging-derived hindered fraction of restricted isotropic diffusion (NII-RF) between the healthy control (n = 67) and ME/CFS (n = 67) participant groups without controlling for confounding factors. Results are displayed in Montreal Neurological Institute (MNI) 152 standard space based on the reference FSL_HCP1065 fractional anisotropy 1x1x1mm standard-space image. The top row shows the results from six different axial slices, where the *z*-coordinates in MNI space from left to right are *z* = -12 mm, -2 mm, 8 mm, 18 mm, 28 mm, and 38 mm, respectively. The middle row shows the results from six different coronal slices, where the *y*-coordinates in MNI space from left to right are *y* = -46 mm, -36 mm, -26 mm, -16 mm, -6 mm, and 4 mm, respectively. The bottom row shows the results from six different sagittal slices, where the *x*-coordinates in MNI space from left to right are *x* = -35 mm, -20 mm, -5 mm, 10 mm, 25 mm, and 40 mm, respectively. Blue-green clusters show the significant decreased NII-RF in ME/CFS participants. The colour bar represents 1-p values (a dimensionless probability measure), with higher values indicating greater levels of statistical significance.


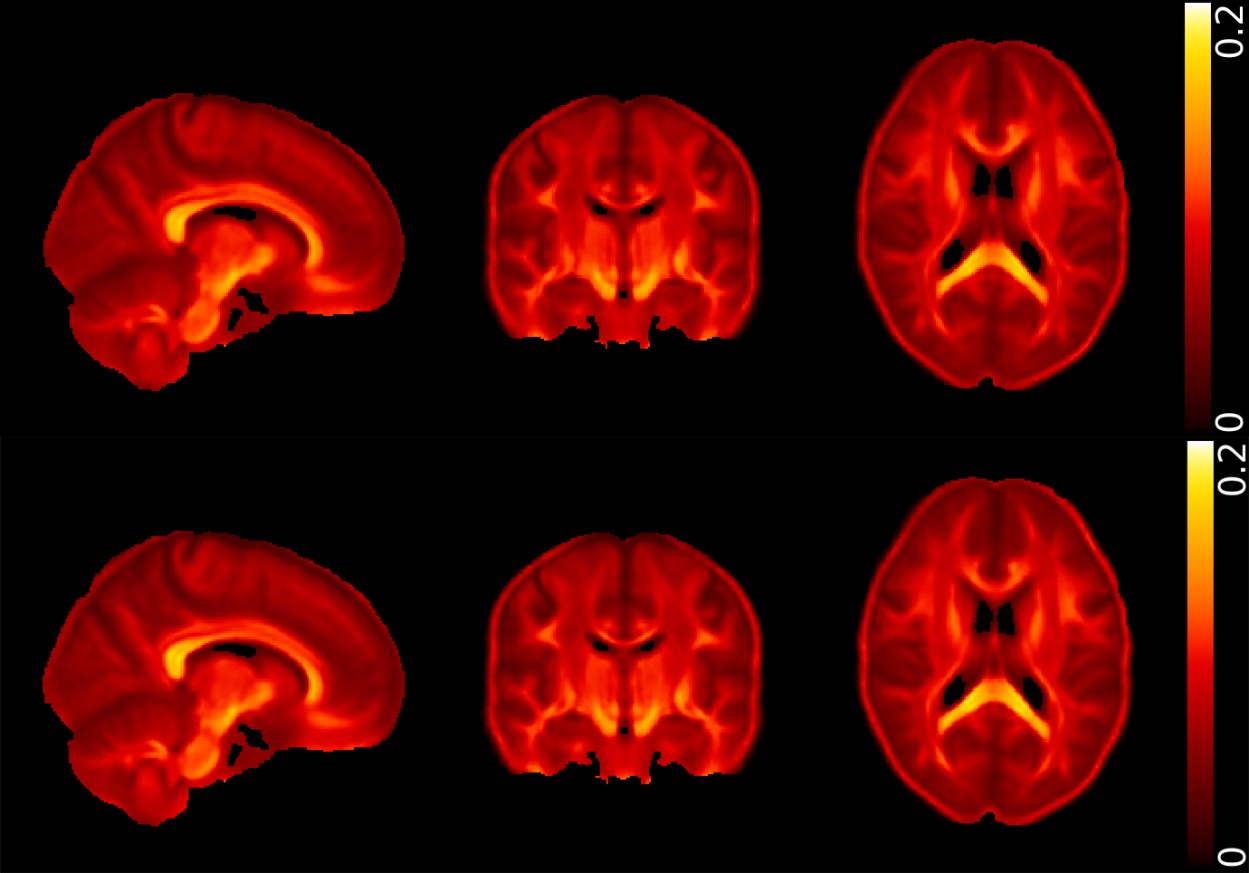


Supplementary Fig. S24: Error map of fitting diffusion MRI signal by solving the diffusion-based neuroinflammation imaging (NII) model using a modified hybrid Nelder-Mead simplex search and particle swarm optimisation algorithm. Results are displayed in Montreal Neurological Institute (MNI) 152 1x1x1mm standard space. The top subfigure shows the normalised mean square error for all ME/CFS patients (n = 67), and the bottom subfigure shows the normalised mean square error for all healthy controls (n = 67). Sagittal (left), coronal (middle), and axial (right) slices are shown at MNI coordinates *x* = 10 mm, *y* = -16 mm, and *z* = 18 mm, respectively. The colour bar represents normalised mean square error values.
